# Cerebral perfusion differences in the visual cortex and fusiform subregions across the psychosis spectrum

**DOI:** 10.3389/fpsyt.2025.1566184

**Published:** 2025-04-29

**Authors:** Jothini Sritharan, Victor Zeng, Jan Petr, Henk-Jan Mutsaerts, Dung Hoang, Nicolas R. Bolo, Elena I. Ivleva, Weiying Dai, Elliot S. Gershon, Sarah K. Keedy, David A. Parker, Rebekah L. Trotti, Jennifer E. McDowell, Brett A. Clementz, Carol A. Tamminga, Godfrey D. Pearlson, Matcheri S. Keshavan, Paulo Lizano

**Affiliations:** ^1^ Advanced Imaging Research Group, Swiss Paraplegic Research, Nottwil, Switzerland; ^2^ Department of Information Technology and Electrical Engineering, Eidgenössische Technische Hochschule (ETH) Zurich, Zurich, Switzerland; ^3^ Department of Psychiatry, Beth Israel Deaconess Medical Center, Boston, MA, United States; ^4^ Division of Translational Neuroscience, Beth Israel Deaconess Medical Center, Boston, MA, United States; ^5^ Institute for Radiopharmaceutical Cancer Research, Helmholtz Center Dresden-Rossendorf, Dresden, Saxony, Germany; ^6^ Department of Radiology and Nuclear Medicine, Location VU University Medical Center, Amsterdam University Medical Center, Amsterdam, Netherlands; ^7^ Amsterdam Neuroscience, Brain Imaging, Amsterdam, Netherlands; ^8^ Department of Psychiatry, Harvard Medical School, Boston, MA, United States; ^9^ Department of Psychiatry, University of Texas Southwestern Medical Center, Dallas, TX, United States; ^10^ School of Computing, Binghamton University, Binghamton, NY, United States; ^11^ Department of Psychiatry, University of Chicago, Chicago, IL, United States; ^12^ Departments of Psychology and Neuroscience, BioImaging Research Center, University of Georgia, Athens, GA, United States; ^13^ Olin Neuropsychiatry Research Center/Institute of Living, Hartford Hospital, Hartford, CT, United States; ^14^ Departments of Psychiatry and Neuroscience, Yale University, New Haven, CT, United States

**Keywords:** arterial spin labeling, cerebral blood flow, V5/MT, fusiform gyrus, psychosis spectrum disorders, cognition

## Abstract

**Background:**

Approximately 50% of individuals with psychosis spectrum disorders (PSD) experience visual hallucinations and deficits in visual processing. Cerebral blood flow (CBF) alterations have been identified in the occipital lobe (OL) and fusiform gyrus (FG) in PSD. However, prior studies neither report on cytoarchitectonic subregions of the OL or FG, nor their correlations with cognition. Moreover, perfusion differences across neurobiologically defined psychosis Biotypes in these regions are not investigated yet.

**Methods:**

ExploreASL and FreeSurfer were used to extract perfusion measures from pseudo-continuous arterial spin labeling scans of visual (hOc1-hOc3v, middle temporal area (MT)) and fusiform (FG2-FG4) subregions in 122 bipolar disorder with psychosis (BP), 179 schizoaffective disorder (SAD), 203 schizophrenia (SZ), and 350 healthy controls (NC), as well as psychosis Biotypes (BT1-3). The data was adjusted for scanner effects using ComBat. Analyses were co-varied for total gray matter CBF. We used R to perform statistical comparisons across PSD and NC and across Biotypes. Partial Spearman correlation was performed between CBF and cognitive measures. Benjamini & Hochberg correction was used to correct for multiple comparisons.

**Results:**

PSD exhibited greater perfusion in MT and FG2 compared to NC. Perfusion significantly differed across psychosis Biotypes in hOc1 but not across diagnostic groups. Higher MT and FG4 perfusion in PSD were associated with worse overall cognitive performance.

**Conclusions:**

Visual and fusiform subregions demonstrate significant perfusion alterations which may indicate neurovascular deficits in PSD. Moreover, these perfusion alterations may contribute to cognitive impairments and visual abnormalities in psychosis.

## Introduction

The visual system is impaired in psychosis spectrum disorders (PSD) and they manifest as perceptual (1), structural (2) and perfusion changes (3). Studies demonstrated that ~50% of individuals with schizophrenia (SZ) experience visual distortions, including altered perception of shape, color, motion, and facial expressions (4–7). Furthermore, it was demonstrated that motion processing is impaired in SZ (8) and that the accuracy of correctly identifying emotional states expressed in human faces is lower in SZ and schizoaffective disorder (SAD) compared to controls, which may affect social cognition (9, 10).

We previously identified brain structural changes which include smaller surface area, thickness, and volume measures in Brodmann area 17 (V1), Brodmann area 18 (V2) and middle temporal area (MT) in individuals with first episode psychosis (2) and PSD compared to controls (11). We also demonstrated that these visual cortical areas are associated with worse negative (2) and positive symptoms (Adhan et al., 2023)^1^, as well as poorer cognition in psychotic disorders (11). With respect to the fusiform gyrus (FG), thinner cortex and smaller cortical surface area in the FG was shown in SZ compared to controls (12). Associations between volume and thickness measures in the FG and a cognitive composite score was found in individuals with SZ (13). These findings emphasize the crucial role that FG plays in SZ.

Basic visual function and facial processing were previously mapped and a new cytoarchitectonic parcellation of the ventral visual stream was developed dividing the occipital lobe (OL) and FG each into four subregions (see **Supplementary Figure S1**) that overlap with retinotopic areas in the brain (14, 15). The associated functions of each region are described in **Supplementary Table S1** (15–31).

Despite the increasing number of studies analyzing the visual system in the context of PSD (32), there is no study to date reporting on perfusion alterations in cytoarchitectonic subregions of the ventral visual stream, which may expand our understanding of visual cortical alterations beyond structural changes. Studies suggested there is increased perfusion in the OL and FG in individuals with PSD (33, 34), while others described decreased perfusion (3, 35). Studies showed lower cerebral blood flow (CBF) in the OL in SZ compared to controls (35–37). In an Arterial Spin Labeling (ASL) study, we previously reported greater grey matter (GM) CBF in the left lateral occipital cortex in bipolar disorder (BD) compared to controls (33). It was shown that higher perfusion in the left lateral occipital cortex was associated with poorer cognition in BD (33). Moreover, it was found that there are sex-specific alterations in the visual cortex in individuals with psychosis (11). Individuals with SZ receiving antipsychotic treatment exhibited greater CBF in the right temporal FG compared with controls (34). A different ASL study found reduced CBF in the FG in all SZ and SAD individuals with positive psychotic symptoms compared to controls (3). Thus, cognitive and symptomatic measures in PSD may be associated with ventral visual stream perfusion in addition to structural changes.

Using a large multi-site dataset from the Bipolar-Schizophrenia Network on Intermediate Phenotypes (B-SNIP) consortium, we aimed to resolve those conflicting findings on perfusion alterations in the ventral visual stream in PSD. The primary goal of this study was to analyze perfusion alterations in cytoarchitectonic subregions of the OL (hOc1-hOc4v), MT, and FG (FG1-FG4) in individuals with psychosis compared to controls. While previous ASL studies following a region of interest (ROI) approach investigated the OL and FG as whole, our study aims at analyzing those regions with greater cytoarchitectonic fidelity which is strongly coupled to retinotopic mapping (15). Secondly, we aim to compare perfusion in the ROIs across psychosis groups using diagnostic groups and neurobiologically defined psychosis Biotypes, which were identified based on electrophysiological and cognitive biomarkers (38). We aimed at determining whether this new grouping is better at differentiating psychosis individuals compared to traditional clinical diagnostic characterization. Lastly, we aimed to investigate correlations between perfusion in the cytoarchitectonic subregions of the OL, FG and MT with cognitive and clinical measures. We expected greater perfusion in OL subregions to be associated with worse symptoms and cognition in individuals with PSD.

## Materials and methods

### Study sample

The study was a retrospective study. Study participants were individuals with psychotic bipolar disorder (BP, n=122), SAD (n=179), SZ (n=203), and healthy controls (NC) (n=350) from the B-SNIP 2 and Psychosis and Affective Research Domains and Intermediate Phenotypes (PARDIP) (non-psychotic bipolar was excluded) study. Study procedures described in (33, 39) were approved by Institutional Review Boards and informed consent was provided by all participants. Participants underwent Structured Clinical Interview for Diagnostic and Statistical Manual of Mental Disorders (DSM)-IV Axis I Disorders (40). Pregnancy, history of head injury with loss of consciousness for more than 10 minutes, intellectual disability, substance use dependence in the past 30 days or history of systemic medical or neurological disorder impacting mood or cognition were exclusion criteria (9). Symptom severity was evaluated using the Positive and Negative Syndrome Scale (PANSS) (41), Young Mania Rating Scale (YMRS) (42), and Montgomery-Åsberg Depression Rating Scale (MADRS) (43). The Global Assessment of Functioning (GAF) score was determined for all participants (44). Participants were asked whether they have ever been a smoker and for the once who answered this question with “Yes”, a follow-up question was asked whether one has smoked in the past 30 days. Additionally, nicotine dependence (very low, low, medium, high, very high) was examined with the Fagerström Test for Nicotine Dependence (FTND) score (45, 46), which was available for 218 participants. A history of visual hallucinations is assessed by the Lifetime of Psychosis Scale (LDPS) (47).

Cognition was assessed based on the Brief Assessment of Cognition in Schizophrenia (BACS) (48). BACS scores were normalized for sex and age to generate z-transformed scores (49). BACS scores were missing from 41 participants.

Furthermore, PSD individuals were assigned a B-SNIP Biotype (BT1-3) according to the method described in (38, 50, 51).The PSD group consisted of 154 BT1, 153 BT2 and 197 BT3. Demographic and clinical characteristics for PSD and NC are displayed in **Table 1** and for diagnostic as well as Biotype groups in **Supplementary Tables S2, S3**, respectively.

**Table 1:** Demographic and clinical measures in individuals with psychosis spectrum disorders and healthy controls.

	NC (n=350)	PSD (n=504)	p-value
Age, Years	33.86 (11.79)	36.84 (11.67)	<0.001
Sex (M/F)	139/211	248/256	0.008
Race (CA/AA/OT)	196/91/63	218/196/90	<0.001
Handedness (R/L/B)	320/25/5	447/46/11	0.411
Lifetime history of being a smoker (Yes/No)	78/270	288/207	<0.001
Smoking in the past 30 days (Yes/No)	22/56	197/91	<0.001
FTND rating (Very low/Low/Medium/High/Very high)	14/4/1/2/1	67/58/28/34/9	0.101
GAF (mean, SD)	84.51 (6.46)	53.59 (13.19)	<0.001
Age of illness onset, Years	–	18.04 (7.54)	–
PANSS Total	–	61.24 (19.68)	–
PANSS General		30.45 (9.39)	–
PANSS Positive	–	15.74 (6.29)	–
PANSS Negative	–	15.01 (6.65)	–
YMRS Total	–	9.11 (7.60)	–
MADRS Total	–	11.24 (10.43)	–
BACS Composite	-0.28 (1.20)	-1.52 (1.37)	<0.001

Values are presented as mean (Standard deviation), n (%), or n.

AA, African American; B, Ambidextrous; BACS, Brief Assessment of Cognition in Schizophrenia; CA, Caucasian; F, Female; FTND, Fagerström Test for Nicotine Dependence; GAF, Global Assessment of Functioning; L, Left; M, Male; MADRS, Montgomery–Åsberg Depression Rating Scale; NC, healthy controls; OT, other; PANSS, Positive and Negative Syndrome Scale; PSD, psychosis spectrum disorders; R, Right; YMRS, Young Mania Rating Scale.

### Image acquisition

Magnetic resonance imaging (MRI) data was acquired during resting-state and participants were asked to keep their eyes open. Seven 3 Tesla scanners were used and pseudo-continuous arterial spin labeling (pCASL) sequences were applied: GE HDx (Athens), GE Discovery MR750 (Boston1, Massachusetts), GE HDxt (Boston2), GE HDxt (Boston3), Philips dSteam Achieva (Chicago), Philips Achieva (Dallas), Siemens Skyra (Hartford) (see **Supplementary Table S4** for scanner parameters, image resolution, and signal-to-noise ratio). Different scanners were used in Boston since there was a hardware and software upgrade during the study.

Additionally, a 1.2 mm isotropic anatomical T1-weighted scan was acquired for each subject, using the Alzheimer’s Disease Neuroimaging Initiative (ADNI)-1/ADNI-2 protocol parameters (52). The sequence type for the Chicago, Dallas and Hartford scanners was MP RAGE, while the Boston and Athens scanners acquired an IR SPGR scan (**Supplementary Table S4**).

### Image pre-processing

Cortical GM CBF maps were prepared using ExploreASL version 1.9.0 (53). Head motion was corrected by an adapted version of the statistical parametric mapping 12 (SPM12) motion correction procedures (53, 54). Partial volume correction was applied by the ExploreASL pipeline (53). ExploreASL’s image processing procedure of bias field correction was omitted in favor of the statistical harmonization method ComBat (https://rdrr.io/bioc/sva/man/ComBat.html). For extracting CBF values in the ROIs, the vcAtlas (see **Supplementary Figure S2**) described in (15) and the MT atlas derived from the FreeSurfer Brodmann Area estimations were transformed from fsaverage to Montreal Neurological Institute (MNI) space using FMRIB Software Library (FSL) (https://fsl.fmrib.ox.ac.uk/fsl/fslwiki/). The transformed atlas was applied on all participants to acquire mean GM CBF for each ventral visual stream subregion (53, 55). FG1 and hOc4v were discarded from further analysis as ExploreASL’s ROI analysis by default skips regions smaller than 1 ml as a sensible cut-off to avoid the analysis of spurious signals coming from too small regions.

For quality control, each CBF image was visually inspected, and 31 images were discarded due to head motion, scanner artifacts blurring the inferior region, labeling failure or missing signal due to inadequate head positioning. Based on a motion parameter estimated by ExploreASL, 17 subjects that deviated more than two standard deviations (SDs) from the mean motion value were removed. Moreover, to address measurement errors, ASL images were removed based on global and lobe-wise GM CBF by site for individuals that deviated beyond four SDs in each of the regions (total n=10). See the **Supplementary Material** for a detailed description of the quality control pipeline.

As seven different ASL acquisition methods were used, significant scanner-dependent differences were introduced to the CBF data. To account for scanner differences, ComBat from the Surrogate Variable Analysis package in R was applied to the total GM CBF and to each of the ventral visual stream ROIs. The perfusion data was normalized before the application of ComBat. Diagnostic group, sex and age were used as covariates to control for differences in the data due to biological variables. ComBat then estimates scanner effects with an empirical Bayes framework and adjusts the data for these effects (56). Significant scanner differences were eliminated in all ROIs after applying ComBat (Before harmonization: **Supplementary Figure S3**, and after harmonization: **Supplementary Figure S4**). Reversing the scaling of the ComBat-adjusted perfusion measures is not possible due to the estimation process, however we applied a simple rescaling and included the main results for the rescaled data in the **Supplementary Material** to aid in providing more biological meaning to the data.

### Statistical analysis

Statistical analyses were performed in R (version 4.1.3). A sensitivity analysis was conducted to examine the moderating effects of demographic and clinical variables on the ROI GM CBF. Outliers in the ROIs deviating more than 4 SDs per site were removed (n=6) and the remaining were winsorized to 3 SDs (n=27). GM CBF in the ROIs was covaried for ComBat-adjusted total GM CBF in order to take potential whole brain perfusion alterations into account and to assess regional specificity (model A) (3, 34). To additionally consider the effect of demographical characteristics, group comparisons were repeated using total GM CBF, sex and age as covariates (model B). As parametric assumptions were violated, two-way analyses of variance (ANOVA) for trimmed means (57) with a group-by-sex design was applied to GM CBF comparisons in the primary (PSD vs. NC) aim. For the secondary aim (diagnostic groups and Biotypes) an ANOVA for trimmed means with a Biotype-by-diagnostic group design was used to compare GM-CBF in the ROIs. Effect sizes were calculated using the explanatory measure of effect size *ξ* (58), with *ξ* =0.10, 0.30, 0.50 representing small, medium, and large effect sizes, respectively (57). Benjamini & Hochberg (BH)-correction was applied to correct for multiple comparisons considering number of regions (7 comparisons) (59, 60). Corrected p-values are denoted by “p_BH_”. The threshold for statistical significance was set at p_BH_=0.05.

Associations between perfusion with cognitive and clinical measures were assessed using partial Spearman correlations due to violation of parametric assumptions. ComBat-adjusted total GM CBF was used as covariate for the scanner-adjusted perfusion measures in model A. For model B, perfusion was covaried for total GM CBF, age and sex. BH-correction was performed accounting for multiple comparisons for cognitive measures (n=7 regions, n=2 groups, n=7 cognitive scores) and for clinical correlations (n=7 regions, n=1 group, n=4 clinical measures). A Fisher’s r to z transformation was conducted to compare correlation coefficients between the two groups. Additionally, a canonical correlation analysis (CCA) was performed between the total GM adjusted perfusion in the ROIs and cognitive scores. Statistical significance of canonical correlation coefficients was assessed using F-Approximation of Wilks’ Lambda (61).

## Results

### Demographic characteristics

Individuals with PSD and NC differed significantly for age, sex, race, GAF, BACS composite score, lifetime history of being a smoker and smoking in the past 30 days, but not for the FTND rating (**Table 1**). The diagnostic groups (excluding NC) showed significant differences in all demographic and clinical variables except for lifetime history of being a smoker and the FTND rating (**Supplementary Table S2**). The Biotype groups and NC differed significantly in age, sex, race, the smoking variables, GAF and BACS composite score, but not in handedness (**Supplementary Table S3**). The Biotypes (excluding NC) differed in all demographic and clinical variables, except for handedness, age of illness onset, YMRS total and MADRS total score.

### Modifying effects on regional GM CBF

Sex showed significant effects on the GM CBF in all ROIs. Age had a significant effect on FG3, hOc1 and MT perfusion, while handedness only displayed effects in FG3. Race, FTND rating and smoking in the past 30 days did not show a significant effect on perfusion in any of the regions.

### Perfusion differences in individuals with PSD

**Figure 1:**
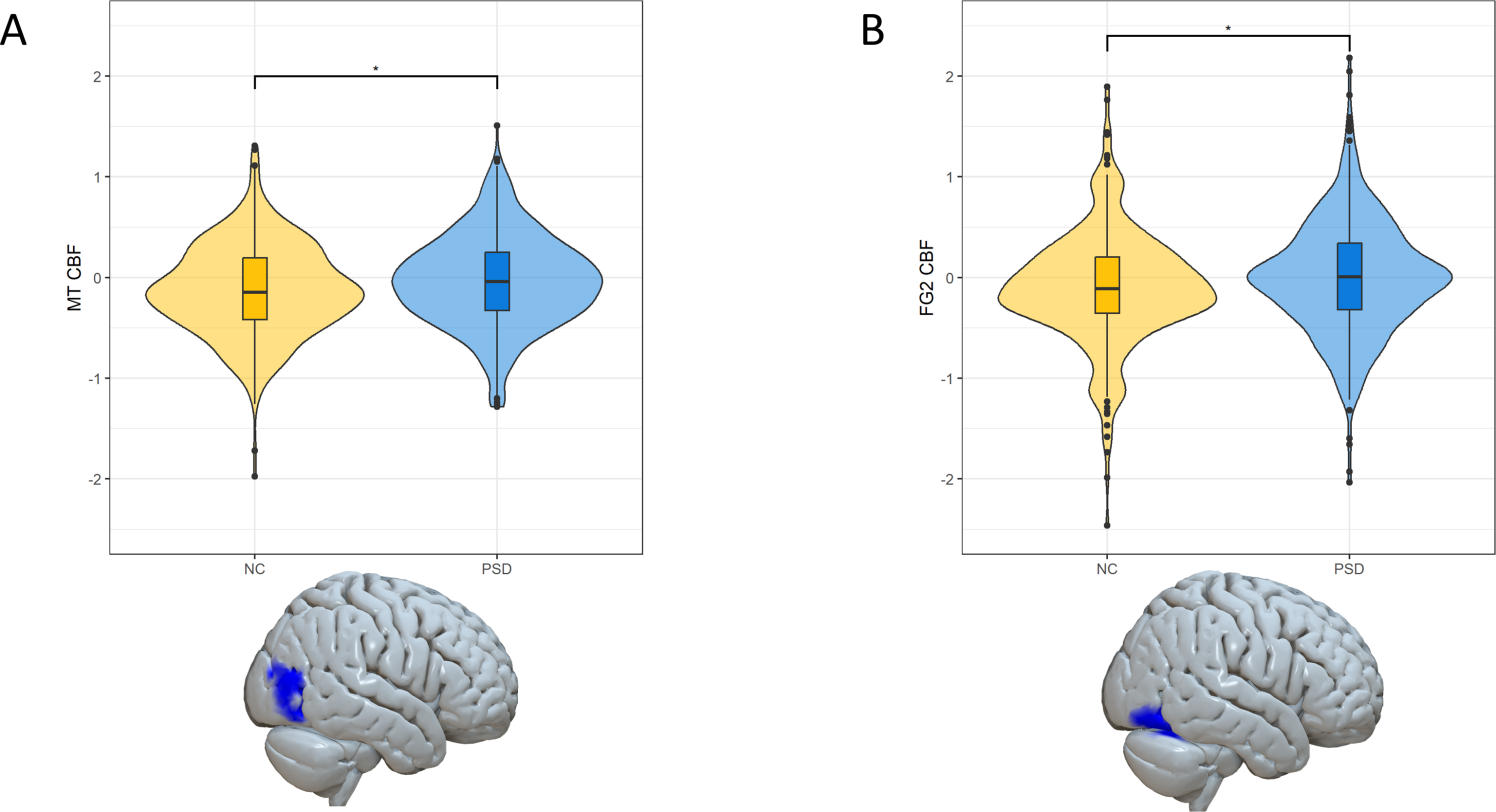
Cerebral blood flow (CBF) differences in MT and FG2. Boxplot with density plots demonstrating group differences between healthy controls (NC) and individuals with psychosis spectrum disorders (PSD) in MT **(A)** and FG2 **(B)**. MT and FG2 CBF were adapted for scanner-differences using ComBat. CBF was covaried for ComBat-adjusted total gray matter (GM) CBF. Panel **(A)** shows significantly higher MT CBF in PSD compared with NC (p_BH_=0.014). Panel **(B)** demonstrates significantly greater FG2 CBF in PSD compared with NC (p_BH_=0.014). * denotes p_BH_<0.05.

Persons with PSD demonstrated greater perfusion in MT (*ξ*=0.142, p_BH_=0.014) and FG2 (*ξ*=0.154, p_BH_=0.014) compared to NC (model A) (**Figures 1A, B**). All other regions did not show significant differences in perfusion between PSD and NC (**Supplementary Table S5**). When covarying perfusion measures for total GM CBF, sex and age (model B), there was a trend of perfusion difference between PSD and NC in MT (*ξ*=0.135, p_BH_=0.056) and FG2 (*ξ*=0.124, p_BH_=0.056) (**Supplementary Table S7**).

**Figure 2:**
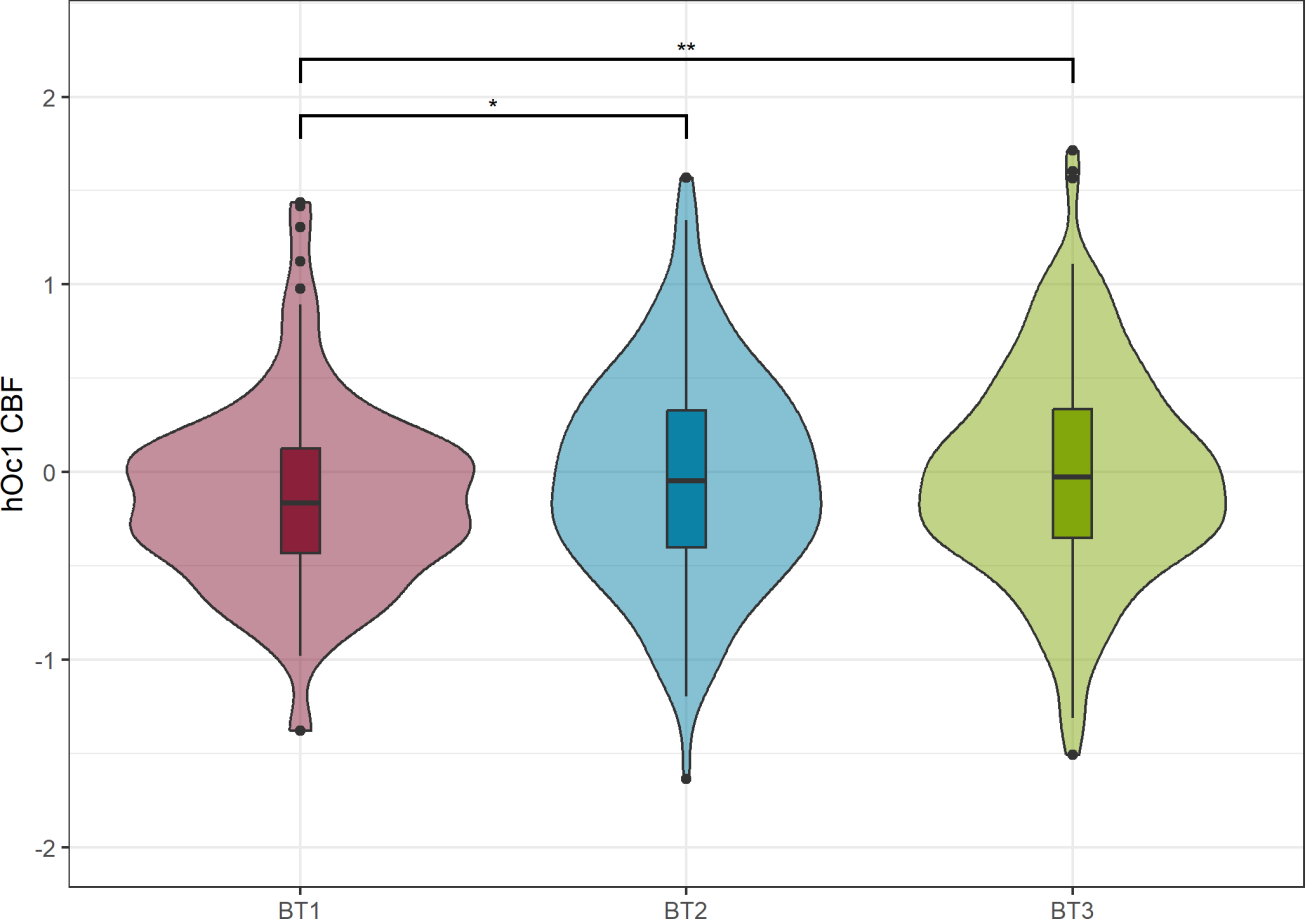
Cerebral blood flow (CBF) differences across Biotypes in hOc1. CBF was adapted for scanner-differences via ComBat and covaried for total gray matter (GM) CBF. There was significantly higher hOc1 CBF in BT2 (p_BH_=0.026) and BT3 compared to BT1 (p_BH_=0.001). Benjamini & Hochberg corrected p-values are denoted with “p_BH_”. * denotes p_BH_<0.05, ** denotes p_BH_<0.01.

The Biotype-by-diagnostic group analysis discarding NC revealed significant perfusion differences across Biotypes in hOc1 (*ξ*=0.155, p_BH_=0.014) (model A). *Post-hoc* tests revealed that Biotypes 2 (*ξ*=0.154, p_BH_=0.026) and 3 (*ξ*=0.198, p_BH_=0.001) showed significantly higher perfusion in hOc1 compared to Biotype 1 (**Figure 2**). There were no significant differences across diagnostic groups and no significant interaction effects between Biotypes and diagnostic groups in any region. The detailed results for all regions are listed in **Supplementary Table S8**. Similarly, for model B Biotype-by-diagnostic group analysis exhibited significant differences across Biotypes in hOc1 (*ξ*=0.132, p_BH_=0.028). Biotype 3 showed higher perfusion compared to Biotype 1 in hOc1 (*ξ*=0.195, p_BH_=0.001). There were no significant diagnostic group differences or interaction effects in any of the regions for model B (**Supplementary Table S9**).

### Associations between perfusion and cognitive and clinical measures

**Figure 3:**
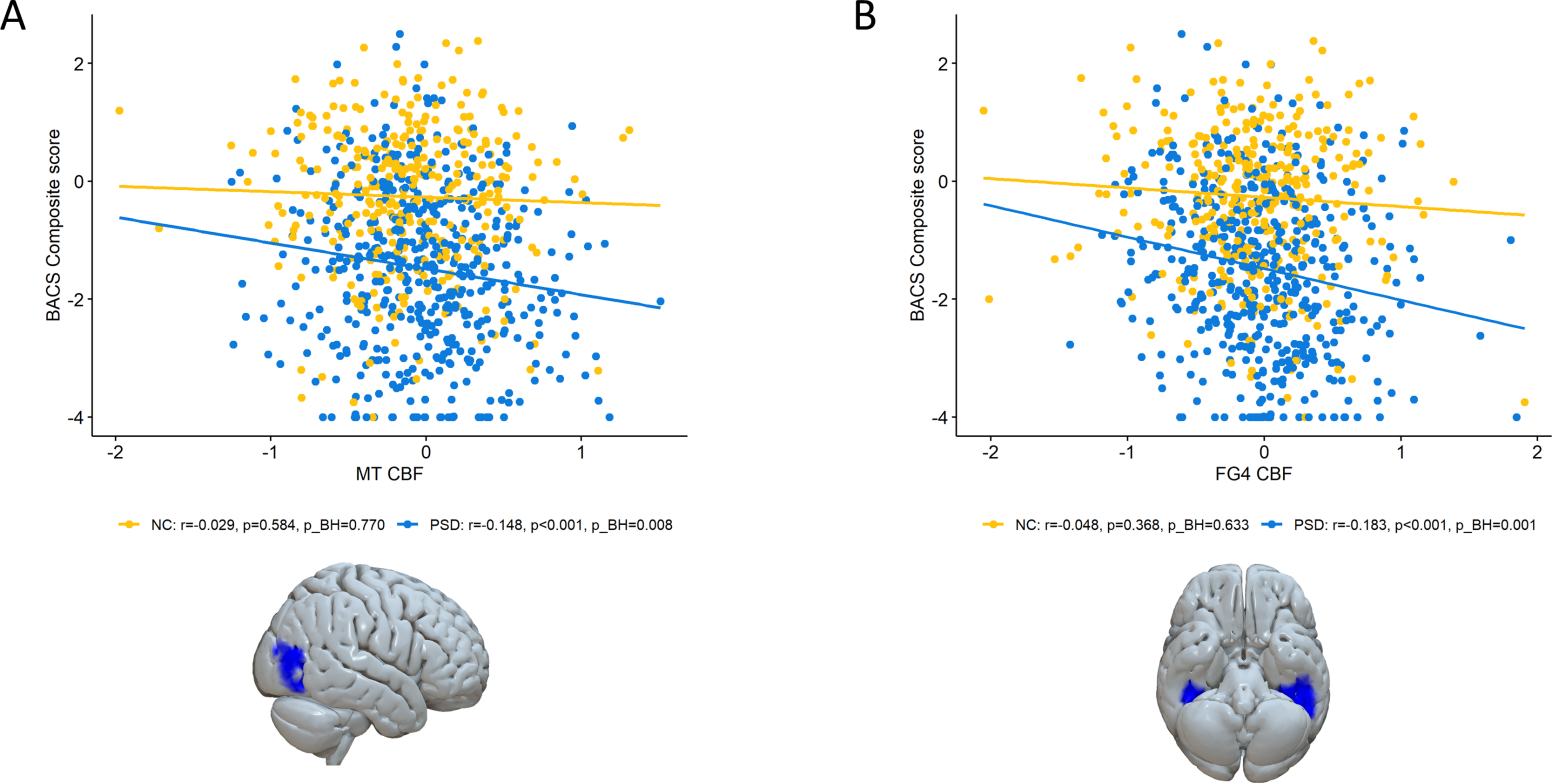
Cerebral blood flow (CBF) correlation with cognitive measures in MT and FG4. Scatter plot in **(A)** showing partial Spearman correlations between scanner-adjusted MT CBF and Brief Assessment of Cognition in Schizophrenia (BACS) composite score in individuals with psychosis spectrum disorders (PSD) and healthy controls (NC). There was a significant correlation between MT CBF and the BACS composite score in PSD (p_BH_=0.008). Scatter plot in **(B)** showing partial Spearman correlations between scanner-adjusted FG4 CBF and BACS composite score in PSD and NC. There was a significant correlation between FG4 CBF and the BACS composite score in PSD (p_BH_=0.001). Total GM CBF was used as covariate for perfusion in all analyses.

In model A, higher MT perfusion in PSD was associated with lower BACS composite score (r=-0.148, p_BH_=0.008) (see **Table 2**, **Figure 3A**). Specifically, greater perfusion in MT significantly correlated with poorer verbal memory (r=-0.188, p_BH_=0.001) and digit sequencing score (r=-0.157, p_BH_=0.006). Higher FG4 perfusion in PSD was associated with lower BACS composite score (r=-0.183, p_BH_=0.001) (see **Table 2**, **Figure 3B**). Greater FG4 perfusion significantly correlated with lower performance in verbal memory (r=-0.149, p_BH_=0.008), symbol coding (r=-0.155, p_BH_=0.006), tower of London (r=-0.158, p_BH_=0.006) and digit sequencing (r=-0.128, p_BH_=0.035) in individuals with PSD (see **Table 2**). There was a negative correlation between hOc2 perfusion and BACS digit sequencing (r=-0.137, p_BH_=0.020). No other region showed significant associations between cognitive measures and perfusion, including those in NC (**Supplementary Table S13**). Regarding the CCA, there were seven canonical dimensions (see **Supplementary Table S15**). Statistical significance was found in the first (p=1.295 10^-7^) and the second (p=0.005) dimension of the CCA. The coefficients suggest that FG4 and MT CBF as well as BACS composite score and verbal memory contribute the most to the first correlation dimension. The second correlation was mostly driven by hOc3v and FG4 CBF as well as the BACS composite and verbal fluency score.

**Table 2:** Correlations between cognitive measures and perfusion in ventral visual stream.

Cognitive Measure	Group	Perfusion Measure	r-value	p-value	p_BH_-value
BACS composite score	PSD	MT CBF	-0.148	6.10 10^-4^	0.008
BACS verbal memory	PSD	MT CBF	-0.188	1.31 10^-5^	0.001
BACS digit sequencing	PSD	MT CBF	-0.157	2.76 10^-4^	0.006
BACS digit sequencing	PSD	hOc2 CBF	-0.137	0.002	0.020
BACS composite score	PSD	FG4 CBF	-0.183	2.21 10^-5^	0.001
BACS verbal memory	PSD	FG4 CBF	-0.149	5.57 10^-4^	0.008
BACS symbol coding	PSD	FG4 CBF	-0.155	3.30 10^-4^	0.006
BACS tower of London	PSD	FG4 CBF	-0.158	2.47 10^-4^	0.006
BACS digit sequencing	PSD	FG4 CBF	-0.128	3.24 10^-3^	0.035

BACS, Brief Assessment of Cognition in Schizophrenia; p_BH_-value=Benjamini & Hochberg corrected p-value; PSD, psychosis spectrum disorders.

Partial Spearman correlations were performed separately in individuals with psychosis spectrum disorders (PSD) and healthy controls (NC) between the cognitive measures (BACS composite score and BACS subscores) and perfusion in the vcAtlas subregions. Scanner-adjusted perfusion measures were covaried for total GM-CBF. BACS scores were already normalized for age and sex. Correction for multiple comparisons was carried out across the seven BACS scores (one composite and six subscores) and perfusion in all regions in the two groups using Benjamini & Hochberg method. Only measures surviving multiple comparison correction are reported in this table.

Comparing the correlation coefficients for the BACS composite score with perfusion in FG4 and MT between PSD and NC showed a trend of difference for FG4 (z=2.106, p_BH_=0.067) and MT (z=1.829, p_BH_=0.067).

For model B, there were significant associations between higher MT perfusion and poorer verbal memory (r=-0.148, p_BH_=0.033) and digit sequencing score (r=-0.139, p_BH_=0.033) in PSD. Greater perfusion in FG4 significantly correlated with worse BACS composite (r=-0.173, p_BH_=0.012) (**Supplementary Table S14**), symbol coding (r=-0.164, p_BH_=0.014), verbal memory (r=-0.138, p_BH_=0.033), digit sequencing (r=-0.137, p_BH_=0.033) and tower of London score (r=-0.142, p_BH_=0.033) in PSD. Greater hOc2 perfusion was associated with worse digit sequencing score (r=-0.131, p_BH_=0.047) for individuals with PSD.

No significant associations were found between perfusion measures with PANSS positive and negative scores, YMRS or MADRS scores for both models (**Supplementary Tables S16, S17**).

## Discussion

In this study, we used pCASL to investigate resting-state CBF and correlations with clinical and cognitive measures in PSD. We demonstrated 1) significantly greater perfusion in MT and FG2 in individuals with PSD compared to NC; 2) significant perfusion differences across Biotypes in hOc1 while BT2 and BT3 showed higher perfusion compared to the BT1; 3) a significant relationship between higher perfusion in both MT and FG4 with poor cognitive performance.

Studies examining perfusion alterations in the ventral visual stream in PSD are limited. The studies that do exist are restricted by small sample sizes and mixed results, ranging from perfusion reductions in the FG in SZ (3) to greater perfusion in the right temporal FG in SZ compared to NC (34), while very few perfusion studies exist examining MT in psychosis (33). Our findings contribute to the literature demonstrating greater perfusion in the ventral visual stream in individuals with psychosis (33, 34). The findings in this study also build upon our work using optical coherence tomography and angiography of the retina in individuals with psychosis, where we demonstrated cytoarchitectural deficits (62–64), as well as retinovascular dysfunction associated with SZ (65, 66). In the latter study, we showed that higher retinovascular measures being associated with worse symptoms and functioning in the early stages of SZ and with lower symptoms and better functioning in the later stages of SZ.

From a functional standpoint, MT is involved in processing visual motion information (27, 67). It was previously shown that individuals with PSD had significantly reduced area, volume and thickness in V1, V2 and MT compared to NC (11). Lesions in MT are linked with abnormal motion perception capabilities, including the perception of velocities (68). With respect to PSD, velocity discrimination (1) and motion processing (8) were significantly impaired in SZ compared to NC. Furthermore, it was shown that SZ individuals exhibited a higher threshold in detecting the direction of motion of a dot pattern relative to NC, which implies a deficit for higher level processing in the visual motion system, involving brain areas such as MT (69). In terms of brain activity, individuals with SZ showed altered activity in the MT compared to NC during visual motion direction and velocity processing tasks (70, 71). These findings emphasize the deficits in determining motion direction and velocity discrimination in PSD, which might be linked to abnormal perfusion in MT observed in this study.

Given the key role of FG2 in face and object recognition (29, 72) and since several studies showed that individuals with PSD exhibit a poorer ability in both discriminating faces (73, 74) and recognizing emotional expressions in faces (9, 75–79), greater CBF in FG2 might contribute to face processing deficits. The hyperperfusion in FG2 in PSD at baseline might be related to neuronal uncoupling, which refers to a disrupted relationship between neural activation, brain metabolism and CBF (80). Neurovascular uncoupling was shown to be present in SZ and SAD (81) and may contribute to the perfusion alterations found in this study. Deficits in facial processing and facial emotion recognition were associated with poor social functioning and problems in social behavior in SZ (82). Abnormal perfusion in the FG might play an important role in social and emotional deficits in PSD. The question of whether there is a correlation between perfusion alterations in FG2 and overall facial processing deficits in PSD requires further investigation.

In PSD individuals there were significant perfusion differences in hOc1 across Biotypes but not across diagnostic groups for both models A and B, which indicates the ability of Biotypes to better discriminate psychosis subtypes in this region from a neurobiological perspective compared to traditional grouping. Furthermore, BT2 showed greater perfusion compared to BT1 in hOc1 (model A), which might be supported by the fact that BT2 is characterized by overactive neural activity (38).

Although it cannot be derived from the current study, what is causing perfusion alterations in PSD remains an open question. Previous studies pointed out that blood-brain barrier (BBB) hyperpermeability, abnormalities of blood vessels and dysfunction of neuroinflammatory responses were linked to SZ (83–88). Another theory proposes that abnormal inflammatory responses in the brain including infections or trauma damage vasculature and disrupt CBF, which harms the BBB (89–91). A consequence could be altered neural signal processing, resulting in the development of SZ (89).

With respect to cognition, we found that higher perfusion in MT was associated with lower BACS composite, digit sequencing and verbal memory score. This finding is in line with a previous ASL study, which found a similar association between perfusion in the occipital cortex and the BACS composite score, the digit sequencing and the verbal memory score in BD (33). Moreover, our results demonstrate associations between higher perfusion in FG4 and poorer BACS performance including the BACS composite, verbal memory, symbol coding, digit sequencing and tower of London score. This finding is supported by a previous functional MRI study with PSD individuals, which found a significant association between loading parameters of an independent component analysis (ICA) element covering the FG with the BACS composite score, tower of London and symbol coding score (92). Despite FG2 having similar functions to FG4, it was not significantly associated with cognition in this study. As FG4 is likely involved in word processing (30), and verbal memory is one of the domains being tested (48), a significant correlation between perfusion in FG4 and verbal memory score adds evidence to the role FG4 may play in word processing. The BACS symbol coding task measures attention and speed of information processing (48). Consequently, our findings suggest that greater perfusion in FG4 is associated with worse attention and lower speed of information processing. In the past, deficits in attention and processing speed in SZ (93, 94) were identified as one of the essential features of cognitive impairment in SZ (95). Our findings emphasize the role of the FG as one of the pathological sites in PSD. A relationship between executive functions, measured by the tower of London task (48), and activity in the FG is lesser known and requires further study.

The translational potential of these findings is evidenced by a recent study conducted by our group where we used lesion network guided high-definition transcranial stimulation to MT and determined that stimulation at this location was a safe, efficacious, and promising approach for reducing general psychopathology via changes in neuroplasticity (96, 97), as well as improving visual hallucinations (98), working memory, verbal fluency and executive functioning (99). Therefore, the perfusion abnormalities found in MT might be an important neural correlate for visual motion and cognitive processing capabilities in psychosis and could be further investigated as a potential target for cognitive rehabilitation in psychosis.

A strength of this study is that significant scanner differences were statistically minimized. Achieving a large sample size by combining data from multiple sites comes at the cost of scanner-dependent differences in the perfusion data. The ASL data in this work has been carefully inspected as part of a thorough quality-check (QC)-pipeline before controlling for scanner differences (see **Supplementary Material**). ComBat successfully reduced scanner-dependent differences while aiming at preserving biological differences (56).

However, there are several limitations to consider. Firstly, the ASL sample being analyzed for this work was not matched for age and sex. These limitations were controlled for by analyzing the interaction effects with sex, regressing out the effect of age and sex as covariates in model B, and comparing model A to B (addition/exclusion of demographic variables). Moreover, all the measured effect sizes were small, which indicates that there are only subtle perfusion differences in the ventral visual stream in psychosis. Another limitation was that mean motion could not be calculated as part of the QC-pipeline for all scanners (see **Supplementary Material**). This was compensated for by visually inspecting all scans. To account for scanner effects in a multi-site study, we applied a scanner harmonization technique, which comes at the cost of data becoming less clinically interpretable in terms of measurement unit due to the normalization and adaptation process of the data. Consequently, a perfect rescaling of the ComBat-adjusted perfusion data to the original measurement unit is not feasible. We implemented a simple rescaling of the data in the **Supplementary Material** to provide more biological meaning to the results. One major restriction of this work is the limited resolution of ASL. Because of the limited resolution two of the original nine structures had to be discarded from the analysis because it was not possible to reliably extract mean CBF values for these small structures. Additionally, this work did not distinguish between left and right hemisphere but rather averaged over the left and right components. Due to this approach, it was not possible to assess lateralized effects which cannot be ruled out in this study. To account for overall perfusion alterations affecting the whole brain, total GM CBF was considered as a covariate in the analysis. Finally, data on visual acuity was not collected from the participants. Future studies should evaluate the relationship between CBF in the visual cortex and visual acuity.

In conclusion, in the largest ASL study to date analyzing perfusion alterations in the ventral visual stream of individuals with psychosis, we demonstrated significant perfusion alterations in visual and fusiform cortex subregions of individuals with psychosis and associations with cognitive impairment. These findings contribute to the growing evidence of alterations manifested in the ventral visual stream in psychosis, emphasizing the visual system as one of the pathological sites in PSD. Significant associations between CBF alterations and cognitive deficits in MT and FG4 may provide specific location information for potential future treatments, targeted at enhancing cognition in psychosis.

## Data Availability

The datasets presented in this article are not readily available because the B-SNIP consortium PI’s have decided to hold off on sharing ASL data for now. Requests to access the datasets should be directed to PL, plizano@bidmc.harvard.edu.

## Footnotes

1 Adhan I, Lizano P, Türkozer HB, Lutz O, Raymond N, Bannai D, et al. Age at psychosis symptom onset impacts visual cortical morphology in psychosis spectrum disorders. *Submit to psychol Med*. (2023).
