# Supplementary material for: Cerebral perfusion differences in the visual cortex and fusiform subregions across the psychosis spectrum
Source: Front Psychiatry. 2025 Apr 29;16:1566184. doi: 10.3389/fpsyt.2025.1566184 (PMC12069394; doi:10.3389/fpsyt.2025.1566184)
Supplement: Supplementary file 1 [file DataSheet1.docx]

**Cerebral Perfusion Differences in the Visual Cortex and Fusiform Subregions Across the Psychosis Spectrum**

**Supplementary Information**

**
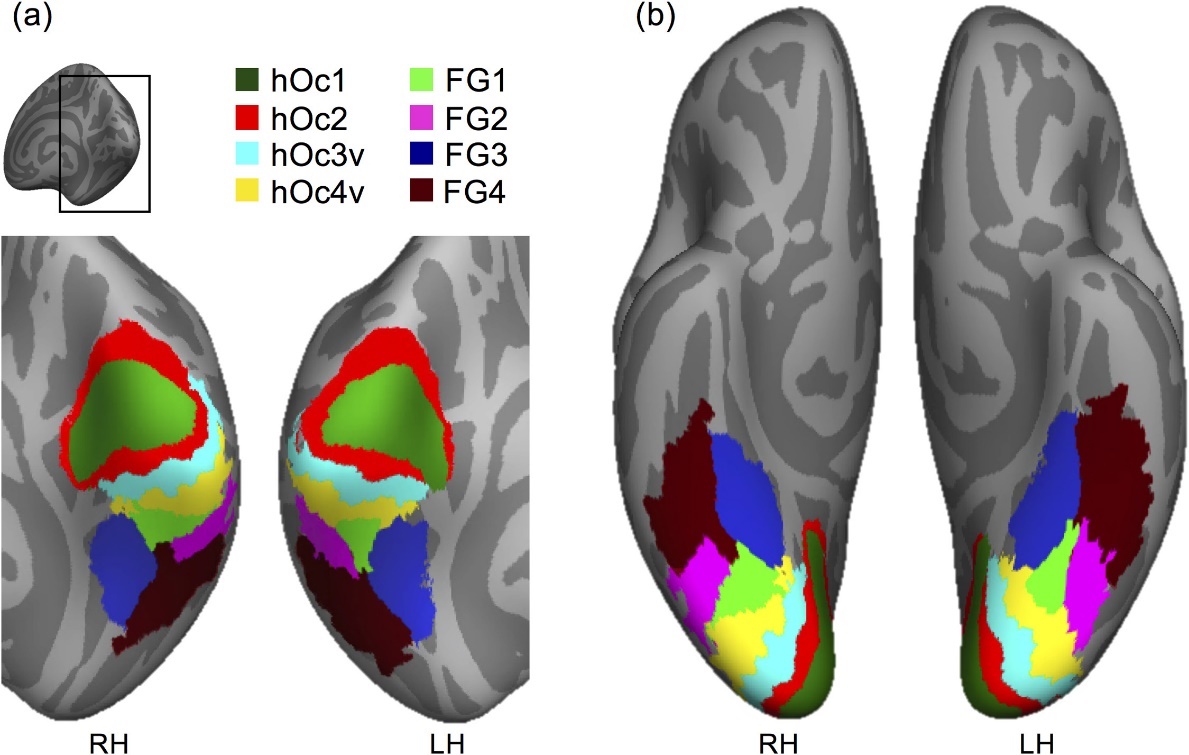
**

**Figure S1.** Cytoarchitectonic regions of the ventral visual stream. The subregions of the fusiform gyrus (FG) and the occipital lobe (hOc) are shown in posterior (a) and ventral view (b). Reprinted with permission from Rosenke et al., "A cross-validated cytoarchitectonic atlas of the human ventral visual stream," NeuroImage, 2018, Volume 170, page 265. © 2017 Elsevier Inc.

**Table S1: Function overview of regions of interest**

| **Structure** | **Function** |
| --- | --- |
| HOc1 | Detecting edges and lines |
| HOc2 | Retrieving color and object information |
| HOc3v | Encoding of color and object orientation |
| HOc4v | Encoding of texture |
| MT | Sensitive to motion |
| FG1 | Recognition of visual objects |
| FG2 | Face perception, visual word processing and recognition of visual objects |
| FG3 | Sensitive to places |
| FG4 | Face perception, visual word processing |


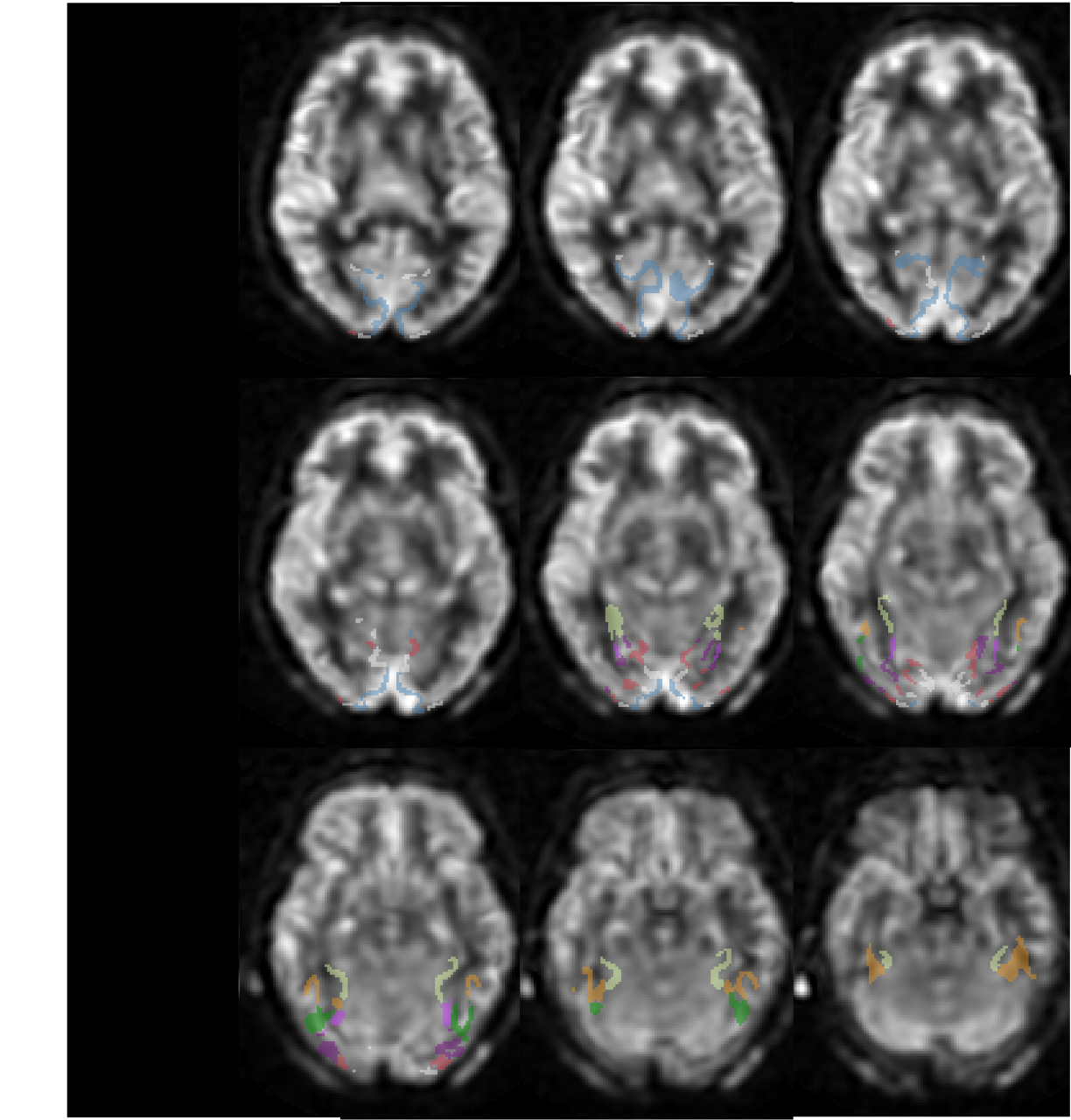


hOc1

hOc2

hOc3v

FG4

FG2

FG3

hOc4v

FG1

**Figure S2.** vcAtlas overlayed on a participant’s cerebral blood flow (CBF) map.

**Table S2: Demographic and clinical measures across diagnostic groups**

|  | NC (n=350) | SZ (n=203) | SAD (n=179) | BP (n=122) | p-value all groups | p-value PSD |
| --- | --- | --- | --- | --- | --- | --- |
| Age, Years | 33.86 (11.79) | 36.99 (11.58) | 38.35 (12.03) | 34.37 (10.96) | <0.001 | 0.014 |
| Sex (M/F) | 139/211 | 126/77 | 77/102 | 45/77 | <0.001 | <0.001 |
| Race (CA/AA/OT) | 196/91/63 | 65/100/38 | 70/72/37 | 83/24/15 | <0.001 | <0.001 |
| Handedness (R/L/B) | 320/25/5 | 171/26/6 | 163/15/1 | 113/5/4 | 0.034 | 0.030 |
| Lifetime history of being a smoker (Yes/No) | 78/270 | 122/77 | 100/76 | 66/54 | <0.001 | 0.489 |
| Smoking in the past 30 days (Yes/No) | 22/56 | 89/33 | 72/28 | 36/30 | <0.001 | 0.022 |
| FTND rating (Very low/Low/Medium/High/  Very high) | 14/4/1/2/1 | 28/27/14/16/3 | 30/21/10/9/2 | 9/10/4/9/4 | 0.158 | 0.337 |
| GAF (mean, SD) | 84.51 (6.46) | 49.94 (12.87) | 53.81 (12.35) | 59.12 (13.02) | <0.001 | <0.001 |
| Age of illness onset, Years | - | 19.54 (7.01) | 16.94 (8.15) | 17.17 (7.06) | - | 0.001 |
| PANSS Total | - | 63.90 (19.06) | 64.87 (19.82) | 51.47 (17.28) | - | <0.001 |
| PANSS General |  | 30.53 (9.15) | 32.30 (9.39) | 27.54 (9.13) | - | <0.001 |
| PANSS Positive | - | 16.79 (6.26) | 17.22 (6.34) | 11.84 (4.44) | - | <0.001 |
| PANSS Negative | - | 16.52 (6.65) | 15.35 (6.73) | 12.04 (5.53) | - | <0.001 |
| YMRS Total | - | 9.31 (7.03) | 10.42 (8.15) | 6.84 (7.16) | - | <0.001 |
| MADRS Total | - | 8.30 (8.28) | 13.43 (10.95) | 12.75 (11.67) | - | <0.001 |
| BACS Composite | -0.28 (1.20) | -1.912 (1.28) | -1.45 (1.34) | -0.95 (1.35) | <0.001 | <0.001 |

**Note:** Values are presented as mean (Standard deviation), n (%), or n.

AA, African American; B, Ambidextrous; BACS, Brief Assessment of Cognition in Schizophrenia; BP, Psychotic bipolar disorder; CA, Caucasian; F, Female; FTND, Fagerström Test for Nicotine Dependence; GAF, Global Assessment of Functioning; L, Left; M, Male; MADRS, Montgomery–Åsberg Depression Rating Scale; NC, healthy control; OT, other; PANSS, Positive and Negative Syndrome Scale; PSD, psychosis spectrum disorders; R, Right; SAD, Schizoaffective disorder; SZ, Schizophrenia; YMRS, Young Mania Rating Scale.

**Table S3: Demographic and clinical measures across Biotype groups**

|  | NC (n=350) | BT1 (n=154) | BT2 (n=153) | BT3 (n=197) | p-value all groups | p-value PSD |
| --- | --- | --- | --- | --- | --- | --- |
| Age, Years | 33.86 (11.79) | 37.19 (11.89) | 38.92 (10.74) | 34.95 (11.95) | <0.001 | 0.006 |
| Sex (M/F) | 139/211 | 91/63 | 63/90 | 94/103 | <0.001 | 0.006 |
| Race (CA/AA/OT) | 196/91/63 | 53/76/25 | 57/68/28 | 108/52/37 | <0.001 | <0.001 |
| Handedness (R/L/B) | 320/25/5 | 134/16/4 | 137/13/3 | 176/17/4 | 0.871 | 0.959 |
| Lifetime history of being a smoker (Yes/No) | 78/270 | 97/54 | 90/58 | 101/95 | <0.001 | 0.044 |
| Smoking in the past 30 days (Yes/No) | 22/56 | 72/25 | 69/21 | 56/45 | <0.001 | 0.002 |
| FTND rating (Very low/Low/Medium/High/  Very high) | 14/4/1/2/1 | 22/23/16/9/2 | 21/24/3/18/3 | 24/11/9/7/4 | 0.007 | 0.017 |
| GAF (mean, SD) | 84.51 (6.46) | 52.18 (12.87) | 51.21 (12.37) | 56.51 (13.56) | <0.001 | <0.001 |
| Age of illness onset, Years | - | 17.35 (6.51) | 19.03 (7.67) | 17.80 (8.13) | - | 0.136 |
| PANSS Total | - | 63.09 (19.60) | 66.60 (20.78) | 55.94 (17.62) | - | <0.001 |
| PANSS General | - | 30.75 (9.37) | 32.18 (10.18) | 28.96 (8.58) | - | 0.008 |
| PANSS Positive | - | 16.38 (6.38) | 17.62 (6.25) | 13.90 (5.76) | - | <0.001 |
| PANSS Negative | - | 15.88 (6.98) | 16.80 (7.19) | 13.04 (5.39) | - | <0.001 |
| YMRS Total | - | 9.61 (7.84) | 9.48 (8.02) | 8.46 (7.07) | - | 0.312 |
| MADRS Total | - | 10.86 (10.42) | 9.93 (10.10) | 12.45 (10.59) | - | 0.087 |
| BACS Composite | -0.28 (1.20) | -1.80 (1.17) | -2.33 (1.20) | -0.69 (1.17) | <0.001 | <0.001 |

**Note:** Values are presented as mean (Standard deviation), n (%), or n.

AA, African American; B, Ambidextrous; BACS, Brief Assessment of Cognition in Schizophrenia; BP, Psychotic bipolar disorder; CA, Caucasian; F, Female; FTND, Fagerström Test for Nicotine Dependence; GAF, Global Assessment of Functioning; L, Left; M, Male; MADRS, Montgomery–Åsberg Depression Rating Scale; NC, healthy control; OT, other; PANSS, Positive and Negative Syndrome Scale; PSD, psychosis spectrum disorders; R, Right; SAD, Schizoaffective disorder; SZ, Schizophrenia; YMRS, Young Mania Rating Scale.

**Table S4: Scanner specifications**

| Site | Athens,  GA | Boston1,  MA | Boston2,  MA | Boston3,  MA | Chicago,  IL | Dallas,  TX | Hartford,  CT |
| --- | --- | --- | --- | --- | --- | --- | --- |
| Number of subjects | 185 | 88 | 32 | 49 | 193 | 145 | 162 |
| Manufacturer | GE | GE | GE | GE | Philips | Philips | Siemens |
| Model | Signa HDx | Discovery MR750 | Signa HDxt | Signa HDxt | Achieva | Achieva | Skyra |
| Study | BSNIP2 | BSNIP2 | PARDIP | BSNIP2 | BSNIP2 | PARDIP/  BSNIP2 | PARDIP/  BSNIP2 |
| Readout | 3D stack of spirals | 3D stack of spirals | 3D stack of spirals | 3D stack of spirals | 2D EPI | 2D EPI | 3D GRASE |
| Repetition time (ms) | 4580 | 4676 | 5000 | 4600 | 4700 | 4620 | 3690 |
| Echo time (ms) | 9.80 | 10.50 | 7.04 | 4.88 | 12.65 | 12.70 (PARDIP)  13.56 (BSNIP2) | 33.78 |
| Labeling duration (ms) | 1425 | 1450 | 2000 | 1450 | 1800 | 1800 | 1500 |
| Post labeling delay (ms) | 1525 | 1525 | 1800 | 1525 | 1800 | 1800 | 1500 |
| Background suppression | On | Off | On | On | Off | Off | On |
| Number of background suppression pulses | 4 | - | 4 | 4 | - | - | 2 |
| Acquisition of M0 image | Yes | Yes | Yes | Yes | No | No | Yes |
| Acquisition duration (min:sec) | 5:40 | 6:00 | 9:00 | 5:40 | 5:38 | 5:32 | 7:00 |
| Flip angle (°) | 155 | 111 | 155 | 155 | 90 | 90 | 120 |
| Slice thickness (mm) | 4 | 4.5 | 4 | 5.4 | 5 | 5 | 3.8 |
| Number of coil elements | 8 | 32 | 8 | 8 | 8 | 8 | 8 |
| Resolution (mm^3^) | 3.75x 3.75 x 4 | 3.906 x 3.906 x 4.5 | 3.75 x 3.75 x 4 | 3.75 x 3.75 x 4.5 | 3.438 x 3.438 x 5 | 3.438 x 3.438 x 5 | 3.75 x 3.75 x 3.8 |
| Signal-to-noise ratio | NA | NA | NA | NA | 0.453 | 0.435 | 0.572 |

CT, Connecticut; GA, Georgia; IL, Illinois; MA, Massachusetts; NA, not available; TX, Texas

**Supplemental Methods**

**Bipolar-Schizophrenia Network on Intermediate Phenotypes (B-SNIP) Biotypes**

The B-SNIP consortium sought to cluster neurobiologically similar individuals across the psychosis spectrum into distinct subgroups, or Biotypes (1). Biomarkers that were functionally and temporally stable were used and they included 44 markers consisting of general cognition, motor inhibitory, saccadic system, and auditory EEG/ERP tasks (2). A Principal Component analysis was applied on the potential biomarkers to identify independent bio-factors (3). Using the bio-factors in a subsequent k-means clustering resulted in three neurobiologically distinct psychosis Biotypes (3,4) that were replicated in a follow up study (2). BT1 was characterized by poor cognition and low neural response to salient stimuli, while BT2 also had poor cognition but an overactive neural response and poor sensory motor inhibition (4). BT3 was nearly normal on all measures (4).

**Quality control**

The raw sample size was n=1136 Arterial Spin Labeling (ASL) scans. Based on the corresponding structural T1 scan, n=21 scans were discarded due to enlarged ventricles, unexpected lesions, or other reasons.

As part of a visual inspection of all ASL scans, several scans were removed due to a scanner artifact that blurred the inferior regions of the brain for the first few scans acquired in the Chicago site (n=9), head motion (n=15), labeling failure (n=4) or missing signal due to incomplete head positioning in the field of view (n=3). Furthermore, mean motion was calculated by ExploreASL for the Philips and Siemens scanners and subjects deviating for more than two standard deviations (SDs) from the sample’s average mean motion value were discarded (n=17). Mean motion was not calculated for GE, as the scanner saves only the mean subtraction image and offline motion correction could not be performed.

Scans in which the total gray matter (GM) or the lobe-wise CBF deviated beyond four standard deviations from the sample’s average were considered as outliers and removed (n=10).

The asymmetry index was calculated using Al = [(R-L)/R+L)] x 100% for raw total GM CBF and lobe-wise CBF. Outliers deviating beyond 4 SDs from the sample’s average were removed (n=34). These instances of asymmetry were most likely caused by unequal distribution of ASL labeling of either hemisphere.

ComBat, a function in R for scanner harmonization, was applied on total GM CBF, all lobe-wise CBF and all regions of interest (ROIs). There were no significant scanner differences in any of the regions after the application of ComBat. Participants whose harmonized total GM CBF deviated beyond four SDs were removed (n=2). The sample size at the end of the Quality control was 1074 participants.

Moreover, participants lacking substantial information such as the scanner type, Biotype, diagnostic group, handedness or who had at least one missing CBF value in any of the seven ventral visual stream regions were removed from the analysis. Visual spot checks were conducted to specifically examine the ventral visual stream regions. In this respect, 100 randomly sampled subjects from both tail ends of the CBF distribution across the seven ROIs were visually inspected in terms of image quality. Five subjects were removed due to inadequate signal and ringing artifacts in the visual areas. The final sample size was 854.

**Group comparisons in the entire occipital lobe and fusiform gyrus**

The group comparison of individuals with psychosis spectrum disorders (PSD) and controls was also conducted for the entire occipital lobe and fusiform gyrus based on the Desikan-Killiany-Tourville atlas (5) using a group-by-sex design for both models A and B. Moreover, the Biotype-by-diagnostic group analysis was conducted for the occipital lobe and fusiform gyrus for both models A and B considering only individuals with PSD.

**Group comparisons excluding participants from “Boston 2”**

As perfusion measures acquired at the site Boston 2 stood out as being different from the other sites before applying ComBat (Figure S3), a group-by-sex design was applied to compare PSD with HC in the ROIs while excluding participants from Boston 2. Perfusion measures were covaried for total GM CBF (model A).

**Group comparisons across diagnostic groups and Biotypes including NC**

Perfusion measures in all ROIs were compared in a diagnostic group-by-sex and a Biotype-by-sex design using model A.

**Group comparisons of PSD individuals with and without hallucinations**

For the comparison of perfusion in PSD with and without visual hallucinations, a lifetime occurrence of visual hallucination-by-sex design was applied for model A.

**Rescaling of ComBat-adjusted perfusion data**

Due to the normalization and estimation process as part of ComBat, perfusion data cannot be interpreted in the original measurement unit of ml/100g/min anymore. In order to aid in providing some biological meaning to the data, we applied a simple rescaling, using means and standard deviations of the original perfusion data, to transform the ComBat-adjusted data closer to the original range of the unadjusted perfusion data. We provide the main results for the rescaled perfusion data at the end of the **supplementary material**.


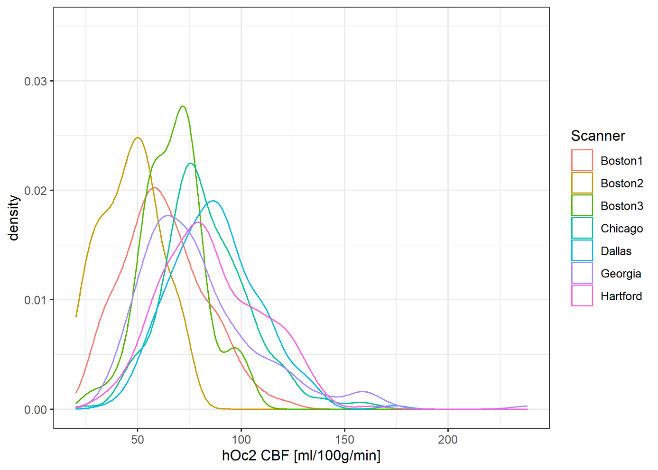

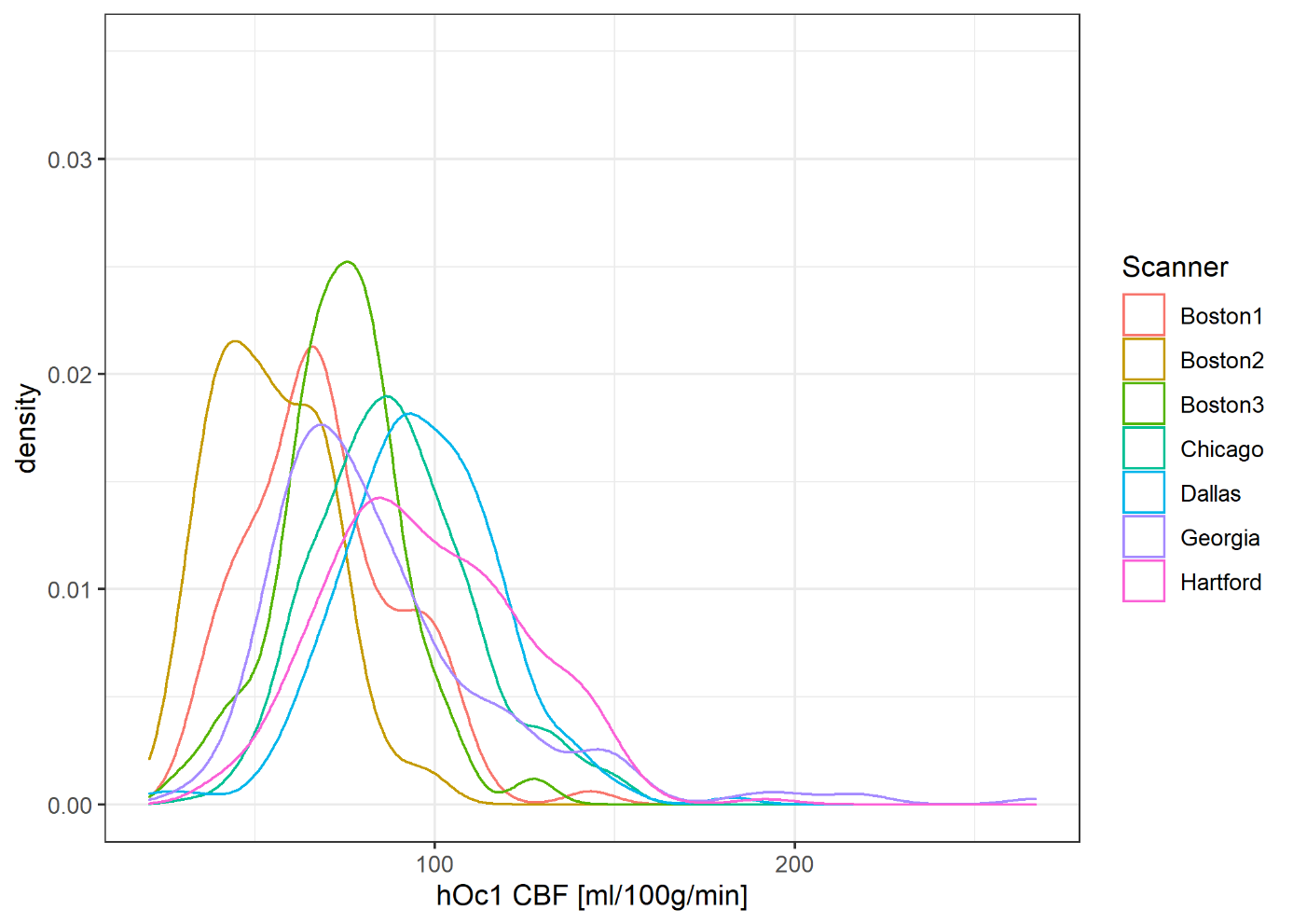

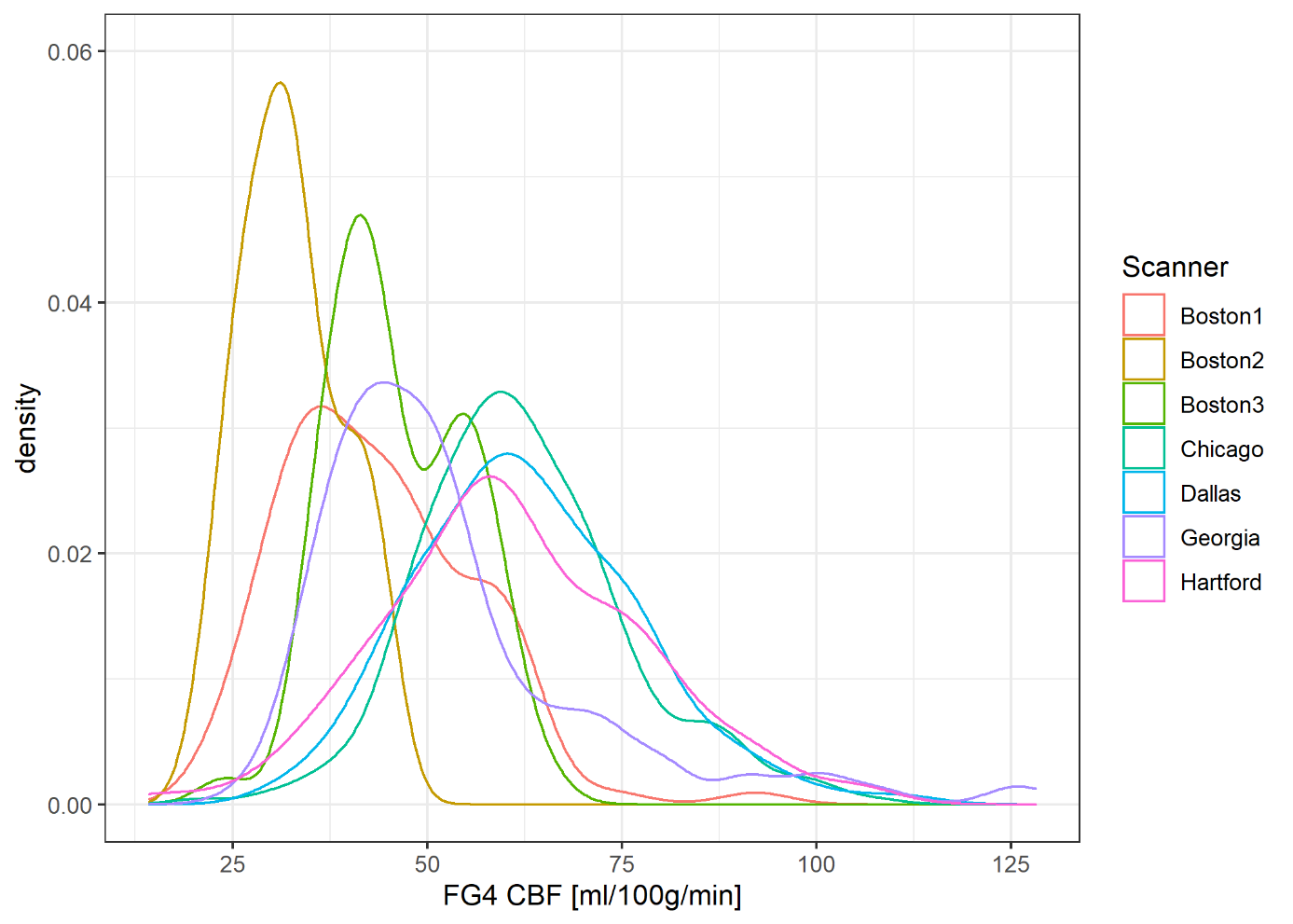

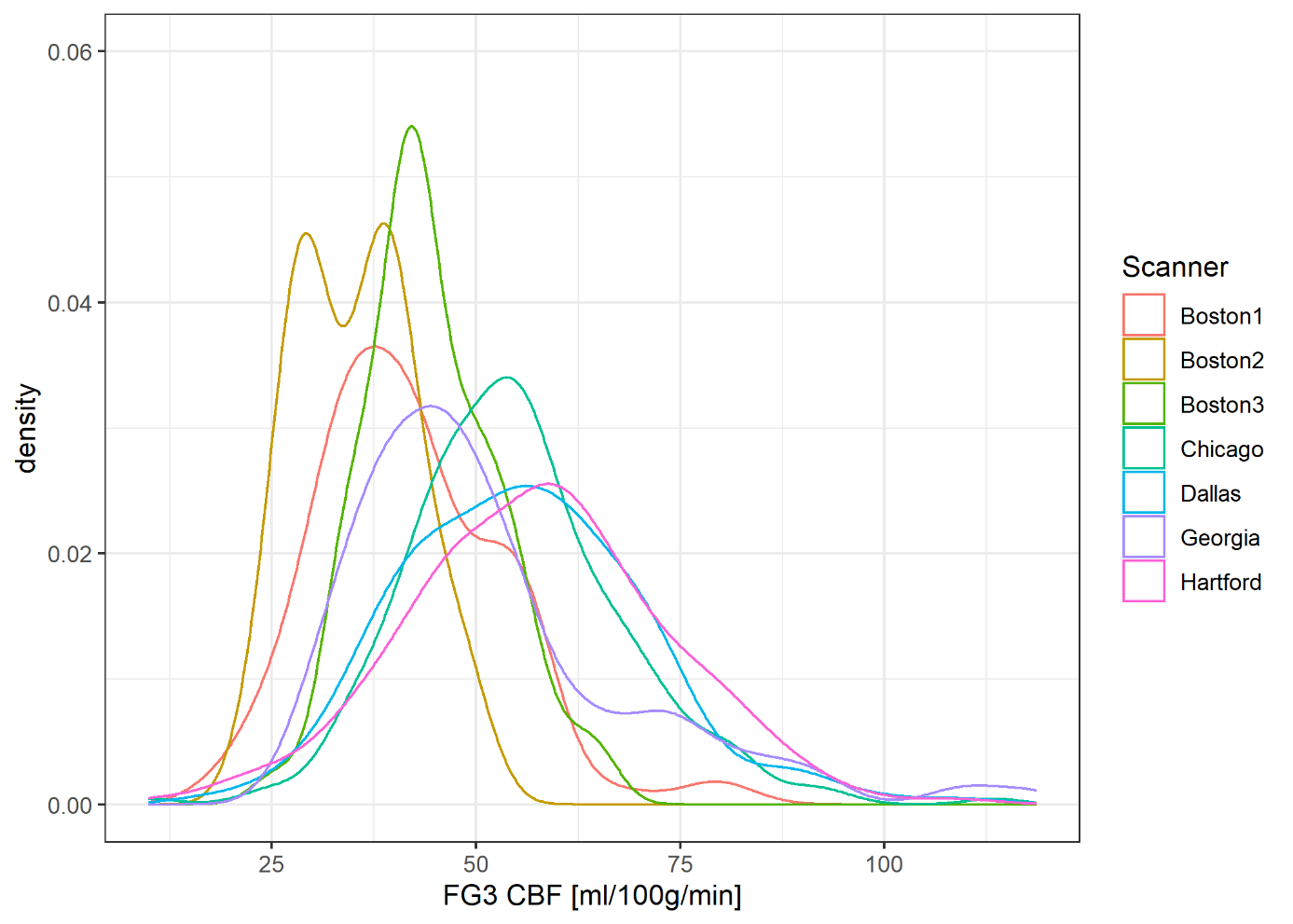

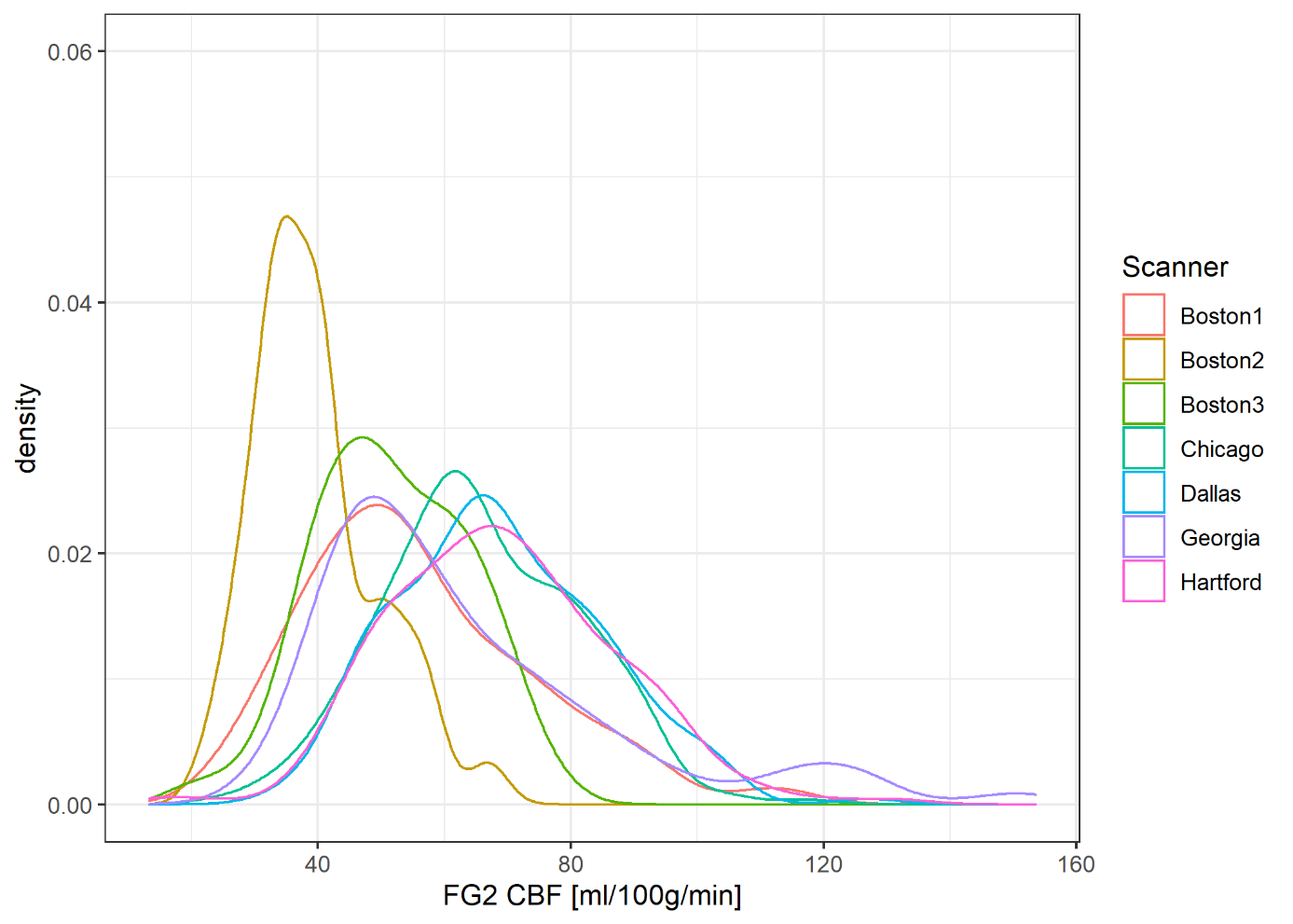

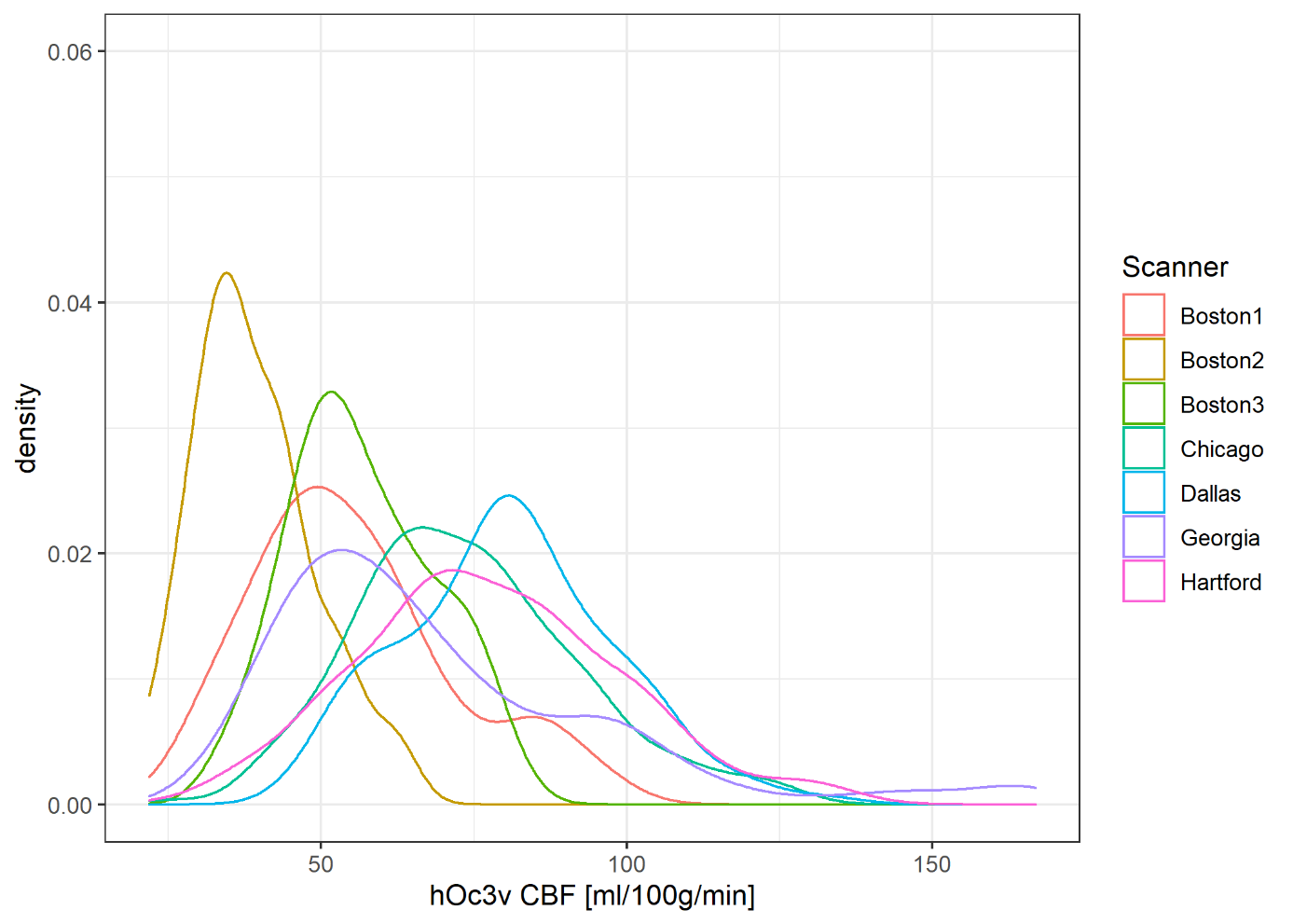

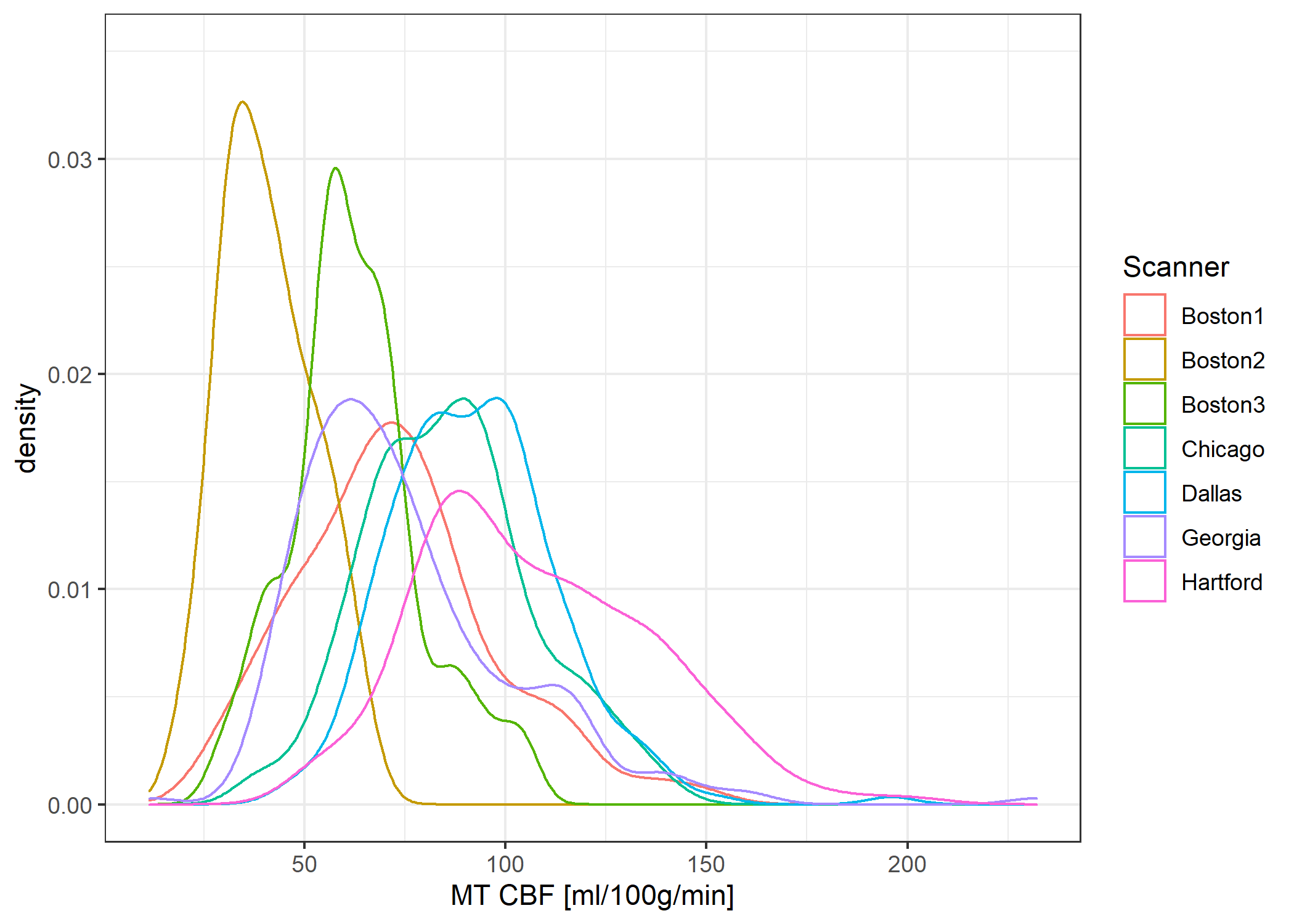


**Figure S3: Perfusion differences by site before outlier removal, winsorization and scanner harmonization.** Density plots showing CBF density per region across scanners before outlier removal, winsorization and ComBat. Different colors represent different scanners. There are significant scanner differences in the CBF data in all regions.


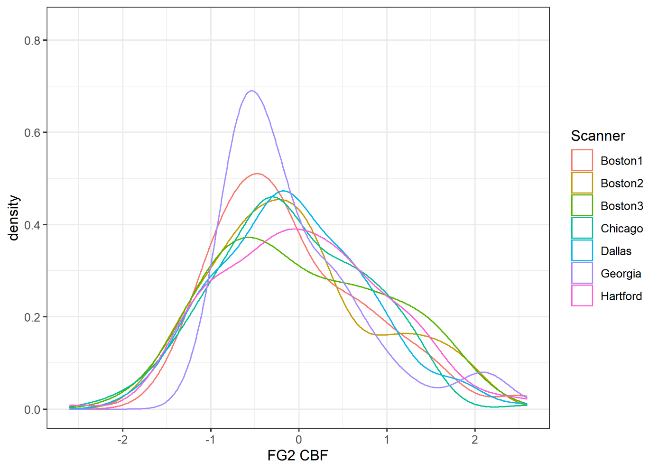

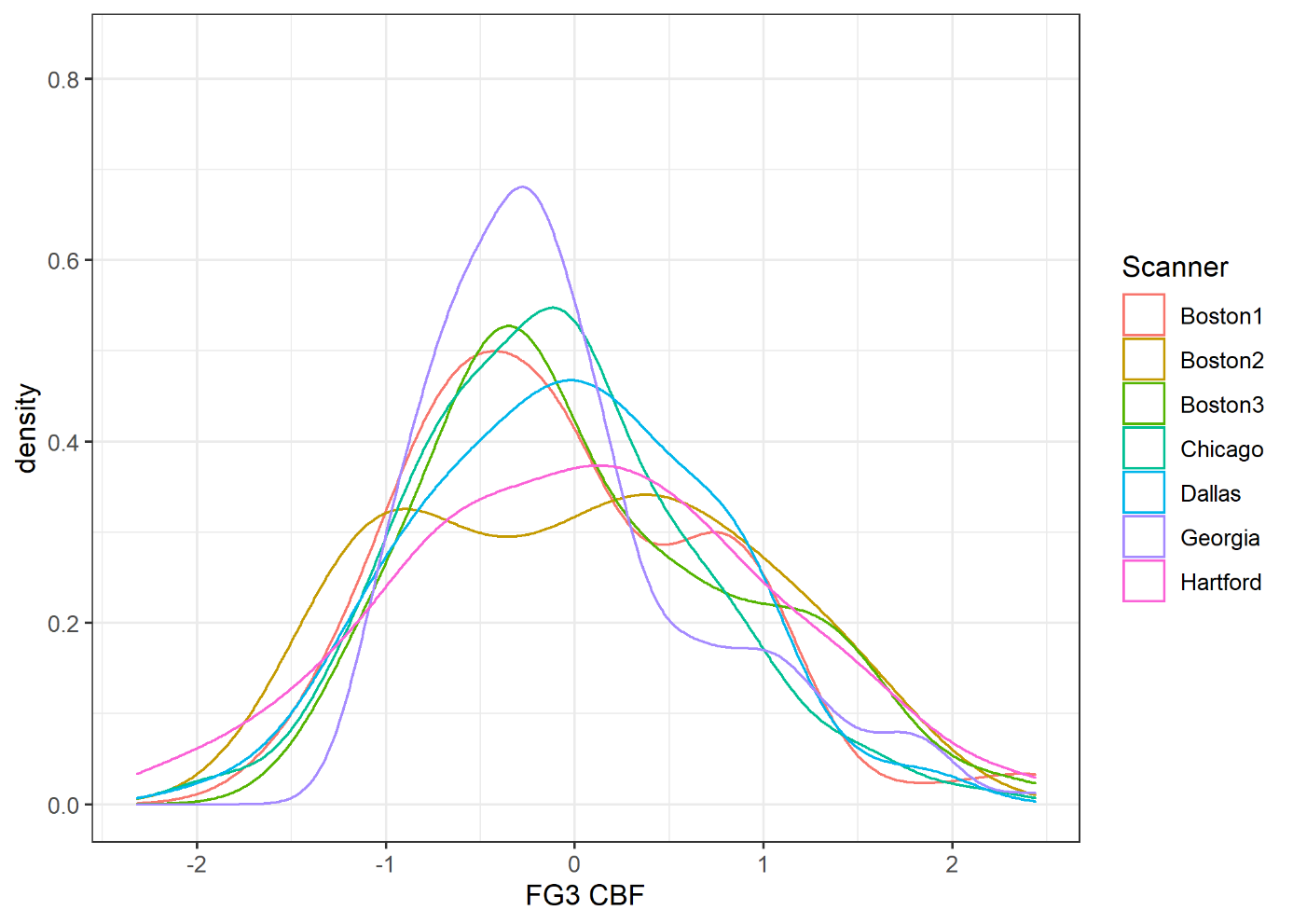

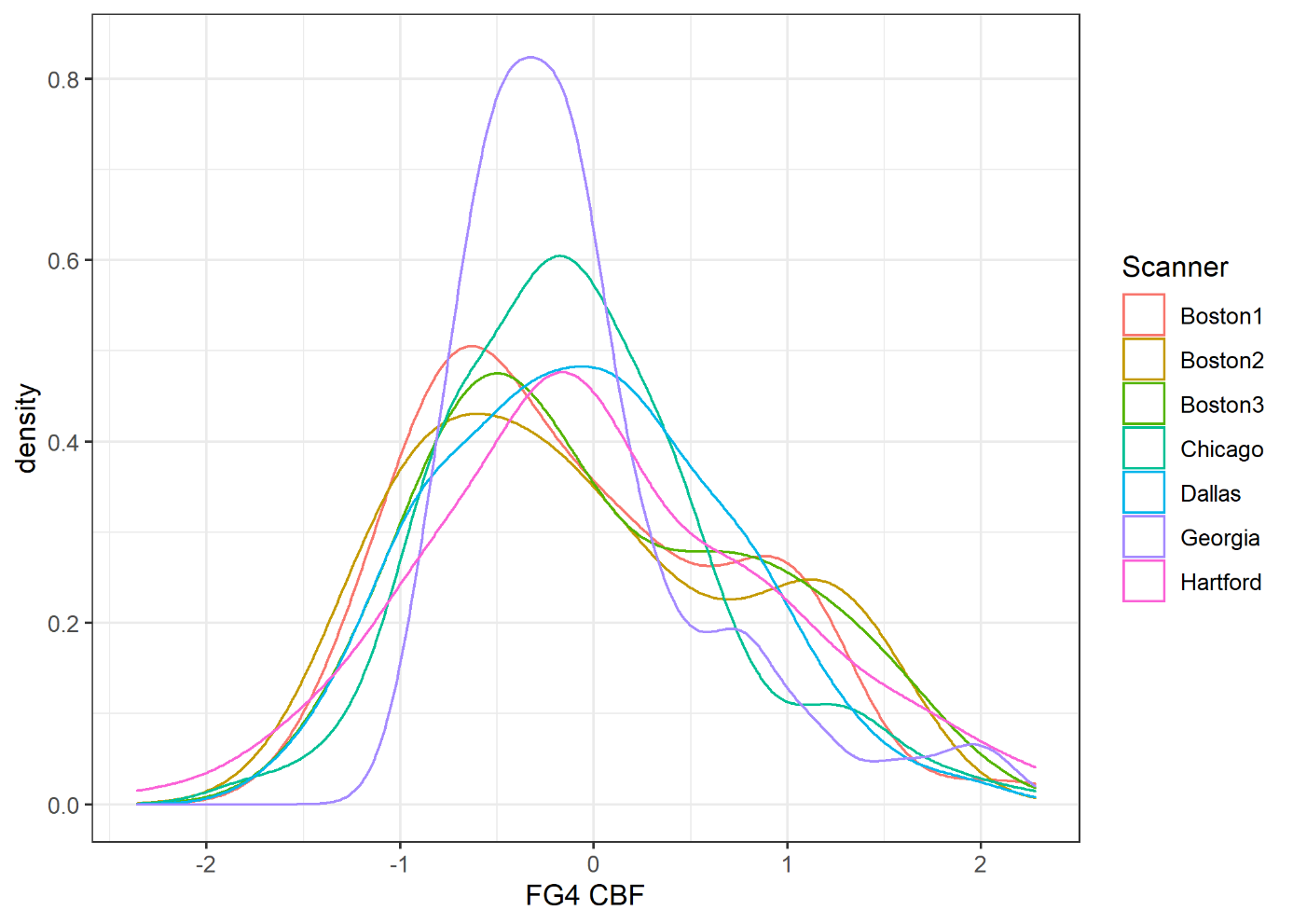

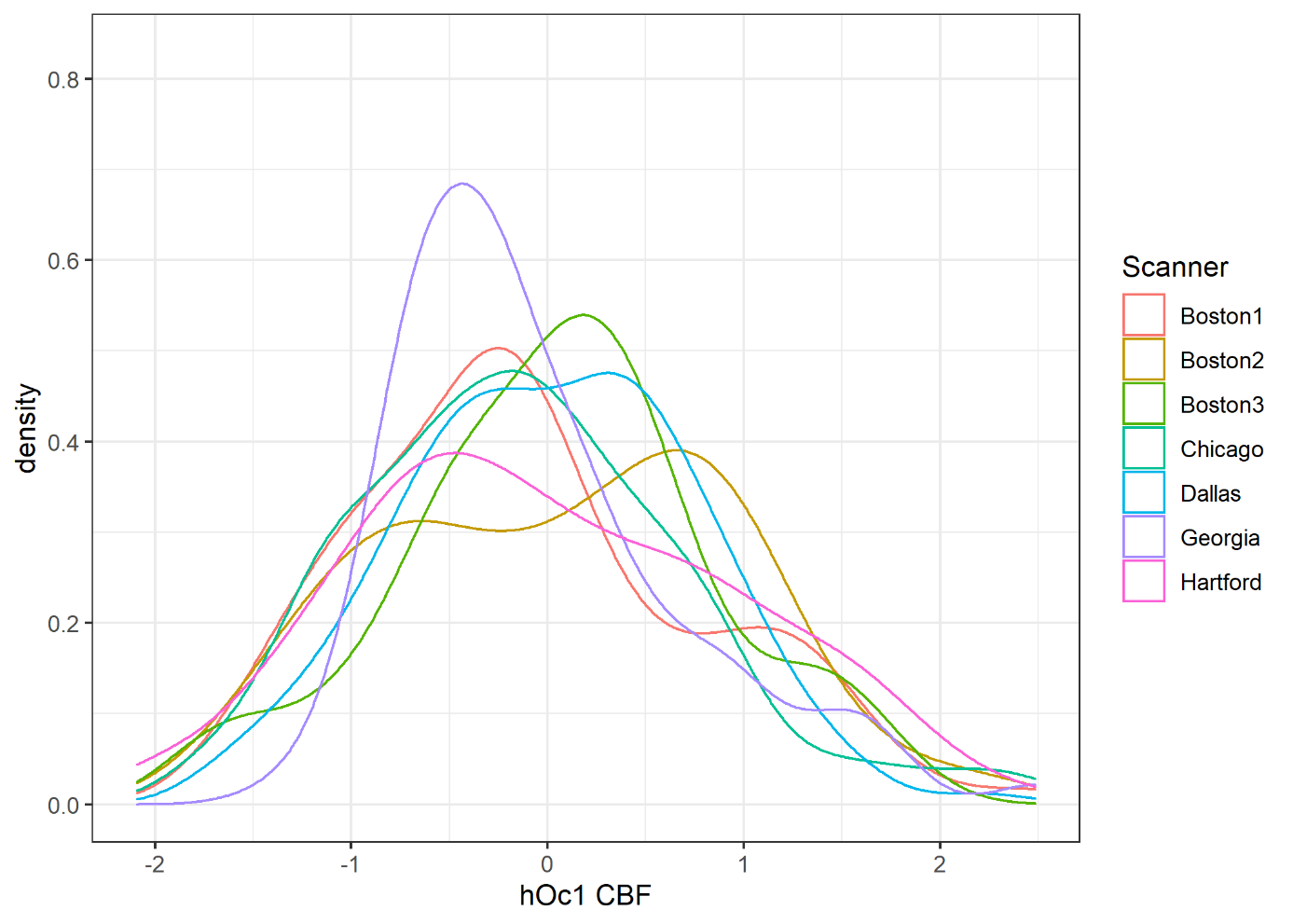

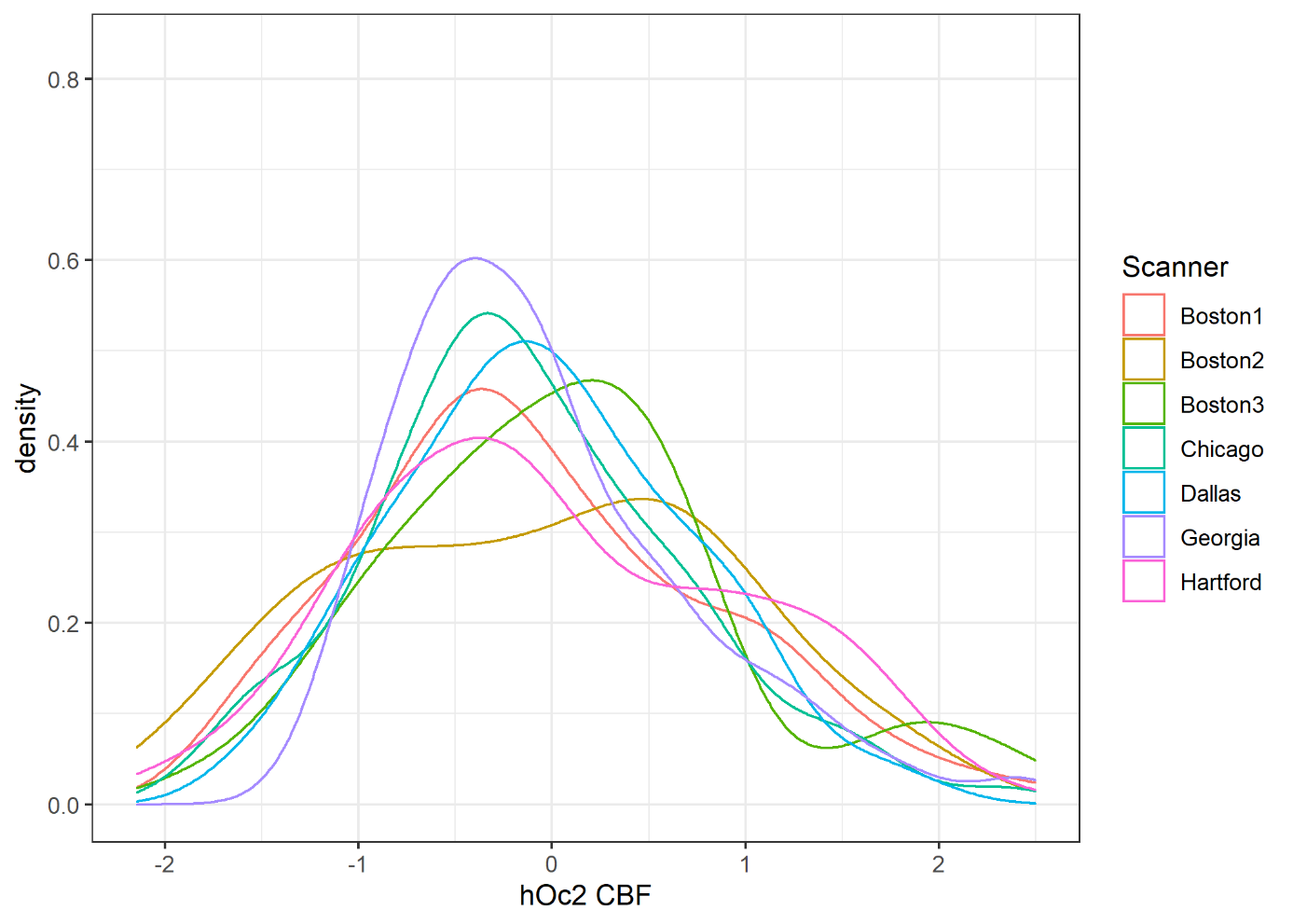

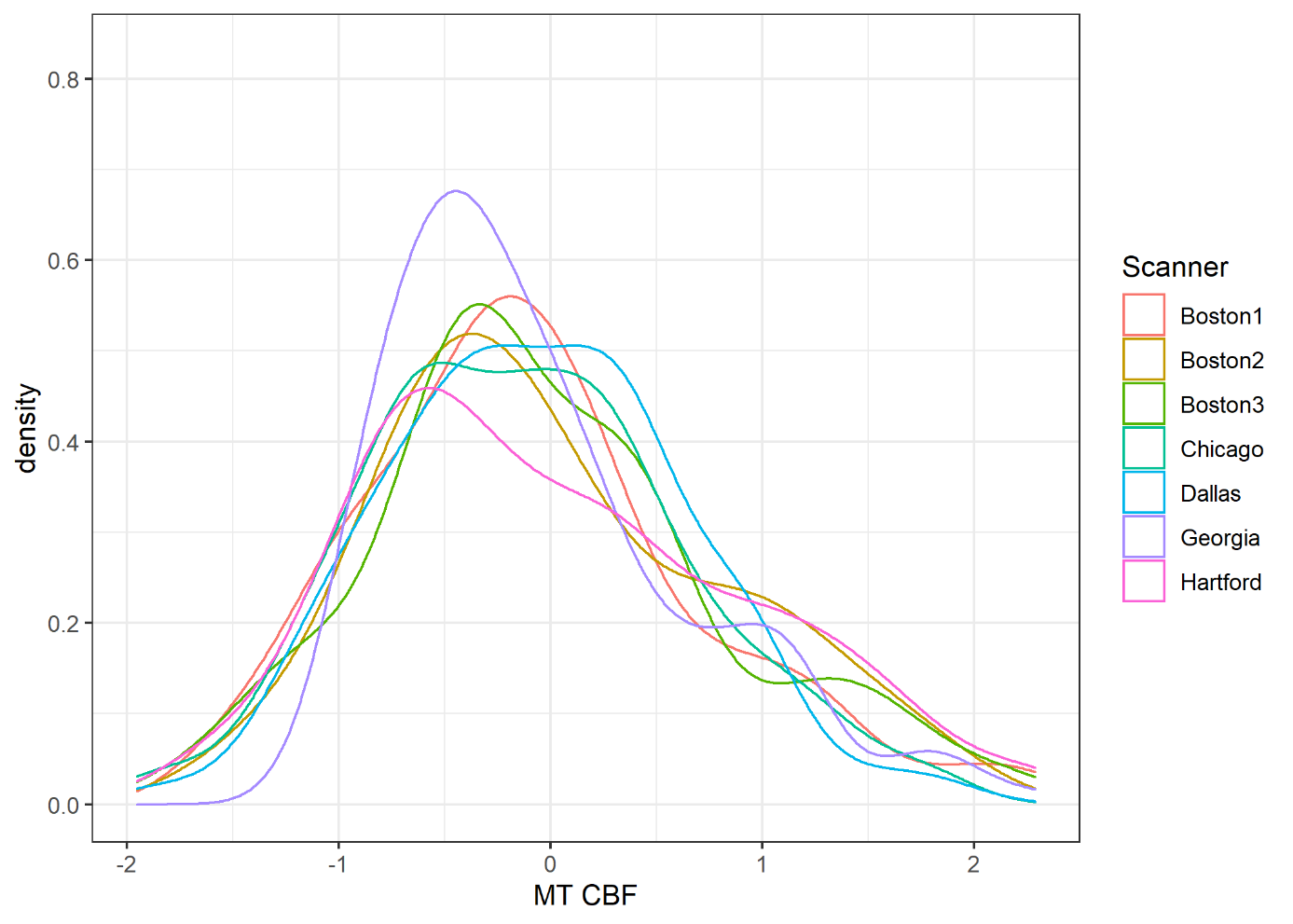

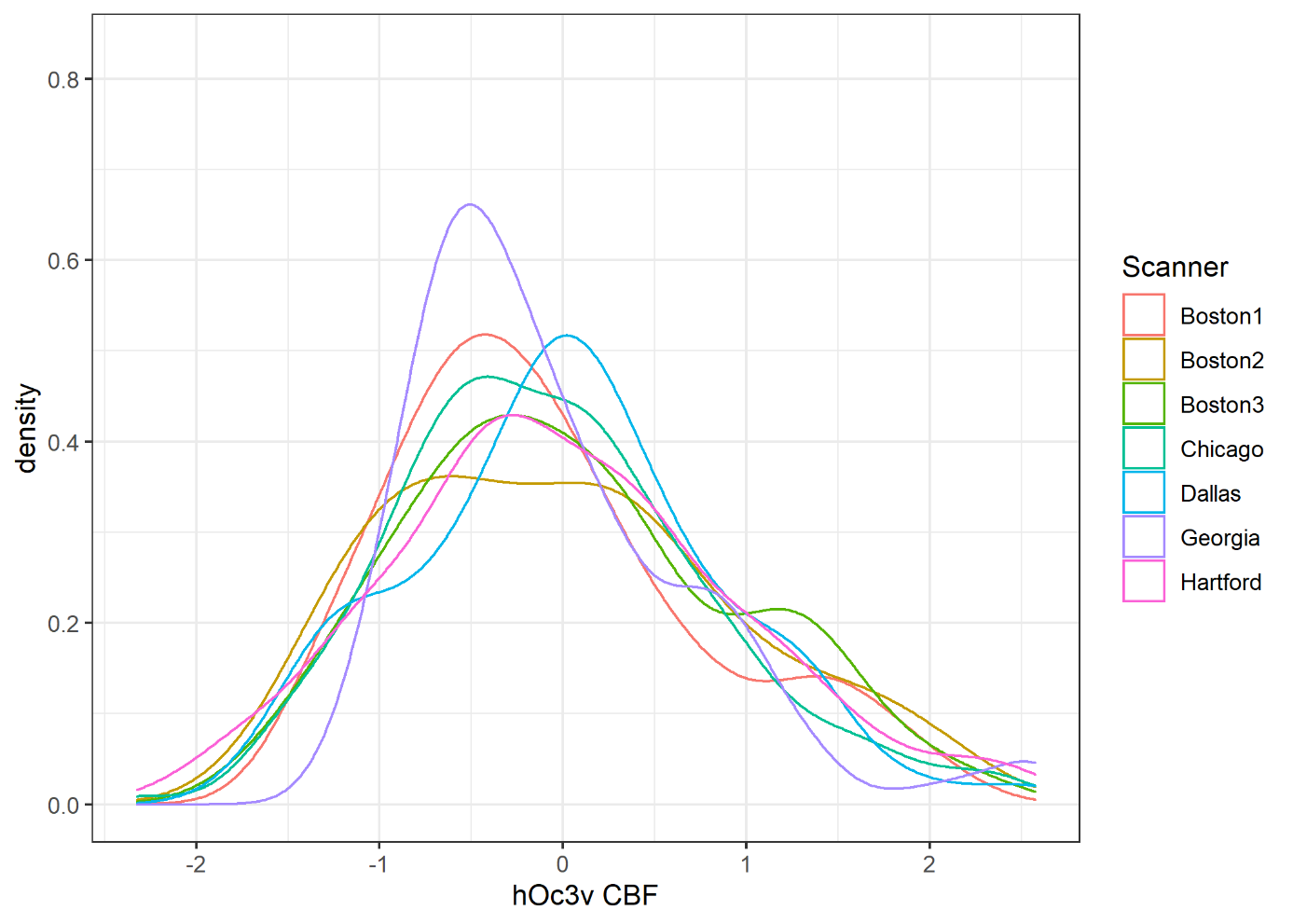


**Figure S4: Perfusion differences by site after outlier removal, winsorization and scanner harmonization.** Density plots showing CBF density per region across scanners after outlier removal, winsorization and ComBat. Different colors represent different scanners. Significant scanner differences in the CBF data were eliminated in all regions.

**Supplemental Results**

1. **Group comparisons in the entire occipital lobe and fusiform gyrus**

There was higher perfusion in individuals with PSD compared to controls in the fusiform gyrus ($\xi$=0.114, p_BH_=0.040) and no significant differences in the occipital lobe ($\xi$=0.015, p_BH_=0.642) (model A). For model B, there was neither a significant difference in the fusiform gyrus ($\xi$=0.084, p_BH_=0.234) nor in the occipital lobe ($\xi$=0.019, p_BH_=0.872).

There were no significant results across Biotypes, diagnostic groups and no interaction effects in the fusiform gyrus and the occipital lobe for both models.

1. **Group comparisons excluding participants from “Boston 2”**

There were significant differences between individuals with PSD and HC in MT ($\xi$=0.156, p_BH_=0.014) and FG2 ($\xi$=0.155, p_BH_=0.014) when excluding participants from the site “Boston 2” (model A) (**Table S6**).

1. **Group comparisons across diagnostic groups and Biotypes including NC**

Comparing perfusion in diagnostic groups including NC, analyses showed perfusion differences in FG2 ($\xi$=0.094, p_BH_=0.049) (model A).

Before correcting for multiple comparisons, FG2 ($\xi$=0.164, p=0.019) and MT ($\xi$=0.167, p=0.012) showed significant differences across Biotypes (model A). However, after correction for multiple comparisons none of those regions remain significant.

Detailed results for the diagnostic group comparison can be found in **Table S10** and for the Biotype group comparison in **Table S11**.

1. **Group-by-sex interactions**

For the analysis, we considered interaction effects of group by sex, because a previous study of our group showed significant group-by-sex interactions for V1 and V2 volume and surface area in individuals with PSD (6).

There were no significant group-by-sex interactions when comparing CBF in the seven ROIs in individuals with PSD and controls while covarying for total GM CBF (model A) (**Table S5**). When comparing perfusion across the single diagnostic groups and NC, there were significant group-by-sex interactions in hOc3 (p_BH_=0.007) and FG3 (p_BH_=0.028) (**Table S10**). There were no significant sex interactions when comparing B-SNIP Biotypes including NC (**Table S11**).

1. **Group comparisons of PSD individuals with and without hallucinations**

Among PSD individuals, 272 individuals had a lifetime history of visual hallucinations and 232 did not. When comparing PSD individuals with and without a history of visual hallucinations (model A), MT showed greater perfusion in individuals with visual hallucinations (ξ=0.148, p=0.014). However, after applying BH-correction, the difference in MT did not remain significant (**Table S12**).

**Table S5: ANCOVA statistics for vcAtlas and MT perfusion measures in PSD vs. NC (model A)**

|  |  |  |  | PSD | | Healthy controls | |
| --- | --- | --- | --- | --- | --- | --- | --- |
| Region | p-value group difference | p_BH_-value group difference | p-value group:sex interaction effect | Trimmed mean | Standard deviation | Trimmed mean | Standard deviation |
| hOc1 | 0.444 | 0.739 | 0.145 | -0.083 | 0.533 | -0.046 | 0.474 |
| hOc2 | 0.874 | 0.874 | 0.340 | -0.071 | 0.534 | -0.060 | 0.534 |
| hOc3 | 0.864 | 0.874 | 0.709 | -0.028 | 0.554 | -0.038 | 0.598 |
| MT | 0.004 | **0.014** | 0.663 | -0.037 | 0.451 | -0.127 | 0.453 |
| FG2 | 0.002 | **0.014** | 0.758 | -0.002 | 0.558 | -0.101 | 0.563 |
| FG3 | 0.528 | 0.739 | 0.141 | -0.024 | 0.504 | -0.041 | 0.520 |
| FG4 | 0.206 | 0.481 | 0.282 | -0.025 | 0.471 | -0.062 | 0.512 |

**Note:** CBF measures were harmonized for scanner differences. Combat-adjusted total gray matter CBF was included as covariate (model A)**.** FG1 and hOc4 had to be discarded because of minimum volume requirements for CBF extraction. Bold values indicate statistical significance at the p_BH_<0.05 level after correction for multiple comparisons.

**Table S6: ANCOVA statistics for vcAtlas and MT perfusion measures in PSD vs. NC discarding participants from site “Boston 2” (model A)**

|  |  |  |  | PSD | | Healthy controls | |
| --- | --- | --- | --- | --- | --- | --- | --- |
| Region | p-value group difference | p_BH_-value group difference | p-value group:sex interaction effect | Trimmed mean | Standard deviation | Trimmed mean | Standard deviation |
| hOc1 | 0.396 | 0.693 | 0.230 | -0.087 | 0.526 | -0.050 | 0.476 |
| hOc2 | 0.760 | 0.883 | 0.641 | -0.075 | 0.530 | -0.054 | 0.531 |
| hOc3 | 0.883 | 0.883 | 0.702 | -0.032 | 0.550 | -0.030 | 0.594 |
| MT | 0.004 | **0.014** | 0.542 | -0.039 | 0.451 | -0.131 | 0.449 |
| FG2 | 0.002 | **0.014** | 0.738 | -0.001 | 0.559 | -0.104 | 0.568 |
| FG3 | 0.584 | 0.818 | 0.190 | -0.026 | 0.506 | -0.038 | 0.523 |
| FG4 | 0.233 | 0.544 | 0.189 | -0.024 | 0.471 | -0.059 | 0.510 |

**Note:** CBF measures were harmonized for scanner differences. Combat-adjusted total gray matter CBF was included as covariate (model A)**.** FG1 and hOc4 had to be discarded because of minimum volume requirements for CBF extraction. Bold values indicate statistical significance at the p_BH_<0.05 level after correction for multiple comparisons.

**Table S7: ANCOVA statistics for vcAtlas and MT perfusion measures in PSD vs. NC (model B)**

|  |  |  |  | PSD | | Healthy controls | |
| --- | --- | --- | --- | --- | --- | --- | --- |
| Region | p-value group difference | p_BH_-value group difference | p-value group:sex interaction effect | Trimmed mean | Standard deviation | Trimmed mean | Standard deviation |
| hOc1 | 0.495 | 0.995 | 0.113 | -0.071 | 0.526 | -0.039 | 0.472 |
| hOc2 | 0.973 | 0.995 | 0.205 | -0.066 | 0.535 | -0.063 | 0.522 |
| hOc3 | 0.995 | 0.995 | 0.909 | -0.038 | 0.541 | -0.036 | 0.588 |
| MT | 0.016 | 0.056 | 0.802 | -0.027 | 0.439 | -0.106 | 0.456 |
| FG2 | 0.012 | 0.056 | 0.707 | -0.008 | 0.554 | -0.088 | 0.548 |
| FG3 | 0.987 | 0.995 | 0.241 | -0.030 | 0.497 | -0.036 | 0.513 |
| FG4 | 0.671 | 0.995 | 0.315 | -0.040 | 0.466 | -0.055 | 0.505 |

**Note:** CBF measures were harmonized for scanner differences. Age, sex and total GM CBF were included as covariates (model B)**.** FG1 and hOc4 had to be discarded because of minimum volume requirements for CBF extraction. Bold values indicate statistical significance at the p_BH_<0.05 level after correction for multiple comparisons.

**Table S8: ANCOVA statistics with Biotype-by-diagnostic group design for vcAtlas and MT perfusion measures excluding NC (model A)**

| Region | p-value  BT  difference | p_BH_-value BT  difference | p-value  dx  difference | p-value  BT:dx interaction effect | p_BH_-value  BT:dx interaction effect |
| --- | --- | --- | --- | --- | --- |
| hOc1 | 0.002 | **0.014** | 0.107 | 0.069 | 0.241 |
| hOc2 | 0.146 | 0.393 | 0.276 | 0.743 | 0.867 |
| hOc3 | 0.170 | 0.393 | 0.516 | 0.881 | 0.881 |
| MT | 0.392 | 0.457 | 0.409 | 0.713 | 0.867 |
| FG2 | 0.485 | 0.485 | 0.311 | 0.201 | 0.469 |
| FG3 | 0.281 | 0.393 | 0.952 | 0.048 | 0.241 |
| FG4 | 0.244 | 0.393 | 0.727 | 0.525 | 0.867 |

**Note:** CBF measures were harmonized for scanner differences. Combat-adjusted total gray matter CBF was included as covariate (model A). BT = Biotype, dx = diagnostic group, p_BH_-value = Multiple comparison corrected p-value with Benjamini & Hochberg method. FG1 and hOc4 had to be discarded because of minimum volume requirements for CBF extraction. Bold values indicate statistical significance at p_BH_<0.05.

**Table S9: ANCOVA statistics with Biotype-by-diagnostic group design for vcAtlas and MT perfusion measures excluding NC (model B)**

| Region | p-value  BT  difference | p_BH_-value BT  difference | p-value  dx  difference | p-value  BT:dx interaction effect |
| --- | --- | --- | --- | --- |
| hOc1 | 0.004 | **0.028** | 0.171 | 0.108 |
| hOc2 | 0.238 | 0.326 | 0.325 | 0.852 |
| hOc3 | 0.058 | 0.203 | 0.303 | 0.750 |
| MT | 0.278 | 0.326 | 0.737 | 0.704 |
| FG2 | 0.441 | 0.441 | 0.422 | 0.619 |
| FG3 | 0.279 | 0.326 | 0.449 | 0.164 |
| FG4 | 0.105 | 0.245 | 0.849 | 0.662 |

**Note:** CBF measures were harmonized for scanner differences. Age, sex and total GM CBF were included as covariates (model B). BT = Biotype, dx = diagnostic group, NC = healthy controls, p_BH_-value = Multiple comparison corrected p-value with Benjamini & Hochberg method. FG1 and hOc4 had to be discarded because of minimum volume requirements for CBF extraction. Bold values indicate statistical significance at the p<0.05 level and p_BH_<0.05 level respectively.

**Table S10: ANCOVA statistics for vcAtlas and MT perfusion measures across diagnostic groups including NC (model A)**

| Region | p-value group difference | p_BH_-value group difference | p-value group:sex interaction effect | p_BH_-value group:sex interaction effect | Trimmed mean (m) per group | Standard deviation (SD) per group |
| --- | --- | --- | --- | --- | --- | --- |
| hOc1 | 0.803 | 0.803 | 0.288 | 0.336 | m_NC_=-0.046  m_SZ_=-0.068  m_SAD_=-0.086  m_BP_=-0.108 | SD_NC_=0.474  SD_SZ_=0.534  SD_SAD_=0.507  SD_BP_=0.568 |
| hOc2 | 0.340 | 0.556 | 0.270 | 0.336 | m_NC_=-0.060  m_SZ_=-0.062  m_SAD_=-0.045  m_BP_=-0.125 | SD_NC_=0.534  SD_SZ_=0.563  SD_SAD_=0.482  SD_BP_=0.561 |
| hOc3 | 0.397 | 0.556 | 0.001 | **0.007** | m_NC_=-0.038  m_SZ_=-0.021  m_SAD_=-0.061  m_BP_=0.017 | SD_NC_=0.598  SD_SZ_=0.564  SD_SAD_=0.521  SD_BP_=0.583 |
| MT | 0.016 | 0.056 | 0.752 | 0.752 | m_NC_=-0.127  m_SZ_=-0.045  m_SAD_=-0.006  m_BP_=-0.064 | SD_NC_=0.453  SD_SZ_=0.452  SD_SAD_=0.476  SD_BP_=0.415 |
| FG2 | 0.007 | **0.049** | 0.184 | 0.322 | m_NC_=-0.101  m_SZ_=-0.043  m_SAD_=0.037  m_BP_=0.010 | SD_NC_=0.563  SD_SZ_=0.562  SD_SAD_=0.561  SD_BP_=0.550 |
| FG3 | 0.662 | 0.772 | 0.008 | **0.028** | m_NC_=-0.041  m_SZ_=-0.024  m_SAD_=-0.007  m_BP_=-0.047 | SD_NC_=0.520  SD_SZ_=0.497  SD_SAD_=0.491  SD_BP_=0.551 |
| FG4 | 0.083 | 0.194 | 0.062 | 0.145 | m_NC_=-0.062  m_SZ_=0.001  m_SAD_=-0.017  m_BP_=-0.070 | SD_NC_=0.513  SD_SZ_=0.477  SD_SAD_=0.479  SD_BP_=0.453 |

**Note:** CBF measures were harmonized for scanner differences. Combat-adjusted total gray matter CBF was included as covariate (model A). p_BH_-value = Multiple comparison corrected p-value with Benjamini & Hochberg method. FG1 and hOc4 had to be discarded because of minimum volume requirements for CBF extraction. Bold values indicate statistical significance at the p_BH_<0.05 level.

**Table S11: ANCOVA statistics for vcAtlas and MT perfusion measures across B-SNIP Biotypes including NC (model A)**

| Region | p-value group difference | p_BH_-value group difference | p-value group:sex interaction effect | Trimmed mean (m) per group | Standard deviation (SD) per group |
| --- | --- | --- | --- | --- | --- |
| hOc1 | 0.089 | 0.156 | 0.298 | m_NC_=-0.046  m_BT1_=-0.157  m_BT2_=-0.052  m_BT3_=-0.037 | SD_NC_=0.474  SD_BT1_=0.481  SD_BT2_=0.554  SD_BT3_=0.549 |
| hOc2 | 0.423 | 0.494 | 0.470 | m_NC_=-0.060  m_BT1_=-0.121  m_BT2_=-0.034  m_BT3_=-0.058 | SD_NC_=0.534  SD_BT1_=0.491  SD_BT2_=0.550  SD_BT3_=0.550 |
| hOc3 | 0.206 | 0.288 | 0.458 | m_NC_=-0.038  m_BT1_=-0.083  m_BT2_=0.041  m_BT3_=-0.039 | SD_NC_=0.598  SD_BT1_=0.542  SD_BT2_=0.531  SD_BT3_=0.578 |
| MT | **0.012** | 0.067 | 0.782 | m_NC_=-0.127  m_BT1_=0.019  m_BT2_=0.003  m_BT3_=-0.074 | SD_NC_=0.453  SD_BT1_=0.442  SD_BT2_=0.455  SD_BT3_=0.455 |
| FG2 | **0.019** | 0.067 | 0.225 | m_NC_=-0.101  m_BT1_=0.009  m_BT2_=-0.034  m_BT3_=0.014 | SD_NC_=0.563  SD_BT1_=0.598  SD_BT2_=0.514  SD_BT3_=0.558 |
| FG3 | 0.727 | 0.727 | 0.494 | m_NC_=-0.041  m_BT1_=-0.010  m_BT2_=-0.039  m_BT3_=-0.026 | SD_NC_=0.520  SD_BT1_=0.447  SD_BT2_=0.476  SD_BT3_=0.561 |
| FG4 | 0.073 | 0.156 | 0.746 | m_NC_=-0.062  m_BT1_=-0.035  m_BT2_=0.047  m_BT3_=-0.073 | SD_NC_=0.513  SD_BT1_=0.454  SD_BT2_=0.459  SD_BT3_=0.493 |

**Note:** CBF measures were harmonized for scanner differences. Combat-adjusted total gray matter CBF was included as covariate (model A). BT1=Biotype 1, BT2=Biotype 2, BT3=Biotype 3, p_BH_-value = Multiple comparison corrected p-value with Benjamini & Hochberg method. FG1 and hOc4 had to be discarded because of minimum volume requirements for CBF extraction. Bold values indicate statistical significance at the p<0.05 level and p_BH_<0.05 level respectively.

**Table S12: ANCOVA statistics for vcAtlas and MT perfusion measures in PSD with and without a history of visual hallucinations (model A)**

|  |  |  |  | PSD with a history of visual hallucinations | | PSD without a history of visual hallucinations | |
| --- | --- | --- | --- | --- | --- | --- | --- |
| Region | p-value group difference | p_BH_-value group difference | p-value group:sex interaction effect | Trimmed mean | Standard deviation | Trimmed mean | Standard deviation |
| hOc1 | 0.043 | 0.149 | 0.414 | -0.041 | 0.523 | -0.130 | 0.541 |
| hOc2 | 0.064 | 0.149 | 0.950 | -0.028 | 0.550 | -0.117 | 0.511 |
| hOc3 | 0.689 | 0.689 | 0.316 | -0.034 | 0.567 | -0.021 | 0.540 |
| MT | **0.014** | 0.098 | 0.891 | 0.010 | 0.450 | -0.089 | 0.444 |
| FG2 | 0.139 | 0.168 | 0.509 | 0.036 | 0.580 | -0.043 | 0.530 |
| FG3 | 0.144 | 0.168 | 0.545 | 0.015 | 0.514 | -0.071 | 0.487 |
| FG4 | 0.121 | 0.168 | 0.107 | 0.013 | 0.480 | -0.070 | 0.457 |

**Note:** CBF measures were harmonized for scanner differences. Combat-adjusted total gray matter CBF was included as covariate (model A). p_BH_-value = Multiple comparison corrected p-value with Benjamini & Hochberg method. FG1 and hOc4 had to be discarded because of minimum volume requirements for CBF extraction. Bold values indicate Upstatistical significance at the p<0.05 level and p_BH_<0.05 level respectively.

**Table S13: Correlations between Brief Assessment of Cognition in Schizophrenia (BACS) scores and perfusion in the visual/fusiform cortex subregions (model A)**

|  | PSD | | | Healthy controls | | |
| --- | --- | --- | --- | --- | --- | --- |
|  | r-value | p-value | p_BH_-value | r-value | p-value | p_BH_-value |
| BACS.composite-hOc1 CBF | -0.031 | 0.476 | 0.707 | -0.076 | 0.160 | 0.462 |
| BACS.composite-hOc2 CBF | -0.066 | 0.127 | 0.433 | -0.022 | 0.684 | 0.808 |
| BACS.composite-hOc3 CBF | -0.031 | 0.470 | 0.707 | -0.114 | 0.043 | 0.193 |
| BACS.composite-MT CBF | -0.148 | $6.10\cdot{10}^{-4}$ | **0.008** | -0.029 | 0.584 | 0.770 |
| BACS.composite-FG2 CBF | -0.052 | 0.233 | 0.538 | 0.001 | 0.992 | 0.992 |
| BACS.composite-FG3 CBF | -0.044 | 0.307 | 0.600 | 0.006 | 0.905 | 0.934 |
| BACS.composite-FG4 CBF | -0.183 | $2.21\cdot{10}^{-5}$ | **0.001** | -0.048 | 0.368 | 0.633 |

**Note:** CBF measures were harmonized for scanner differences. Combat-adjusted total gray matter CBF was included as covariate for the perfusion measures. p_BH_-value = Benjamini & Hochberg corrected p-value. FG1 and hOc4 had to be discarded because of minimum volume requirements for CBF extraction. BACS composite score was corrected for multiple comparisons across number of groups and regions. Bold values indicate statistical significance at the p_BH_<0.05 level.

**Table S14: Correlations between Brief Assessment of Cognition in Schizophrenia (BACS) scores and perfusion in the visual/fusiform cortex subregions (model B)**

|  | PSD | | | Healthy controls | | |
| --- | --- | --- | --- | --- | --- | --- |
|  | r-value | p-value | p_BH_-value | r-value | p-value | p_BH_-value |
| BACS.composite-hOc1 CBF | -0.021 | 0.647 | 0.812 | -0.049 | 0.379 | 0.676 |
| BACS.composite-hOc2 CBF | 0.055 | 0.225 | 0.611 | -0.032 | 0.571 | 0.803 |
| BACS.composite-hOc3 CBF | -0.023 | 0.614 | 0.812 | -0.114 | 0.041 | 0.307 |
| BACS.composite-MT CBF | -0.119 | 0.008 | 0.083 | -0.022 | 0.689 | 0.820 |
| BACS.composite-FG2 CBF | -0.051 | 0.261 | 0.614 | 0.025 | 0.659 | 0.812 |
| BACS.composite-FG3 CBF | -0.048 | 0.295 | 0.628 | -0.014 | 0.803 | 0.886 |
| BACS.composite-FG4 CBF | -0.173 | $1.21\cdot{10}^{-4}$ | **0.012** | -0.043 | 0.438 | 0.740 |

**Note:** CBF measures were harmonized for scanner differences. Combat-adjusted total gray matter CBF, age and sex were included as covariates for the perfusion measures. p_BH_-value = Benjamini & Hochberg corrected p-value. FG1 and hOc4 had to be discarded because of minimum volume requirements for CBF extraction. BACS composite score was corrected for multiple comparisons across number of groups and regions. Bold values indicate statistical significance at the p_BH_<0.05 level.

**Table S15: Correlation coefficients of canonical correlation analysis between cognitive and perfusion measures (model A)**

|  | 1^st^ canonical variate | 2^nd^ canonical variate | 3^rd^  canonical variate | 4^th^ canonical variate | 5^th^  canonical variate | 6^th^ canonical variate | 7^th^ canonical variate |
| --- | --- | --- | --- | --- | --- | --- | --- |
| FG2 CBF | -0.115 | 0.716 | 1.121 | -0.416 | 1.043 | 0.539 | -1.234 |
| FG3 CBF | 0.488 | 0.086 | 1.11 | 0.841 | 0.224 | 1.524 | 1.517 |
| FG4 CBF | -1.378 | 1.206 | -1.056 | 0.42 | -1.178 | -1.583 | -0.136 |
| hOc1 CBF | 0.405 | 0.198 | -0.226 | -0.312 | -1.814 | 1.217 | -1.085 |
| hOc2 CBF | -1.004 | -1.053 | 1.339 | -0.745 | 0.182 | -1.073 | 0.793 |
| hOc3 CBF | 0.069 | -1.202 | -0.806 | 1.45 | 0.421 | -0.081 | -0.599 |
| MT CBF | -1.1 | -0.007 | -1.374 | -0.651 | 0.595 | 1.04 | 0.728 |
| BACS composite | -1.011 | -1.482 | 2.942 | 3.537 | -0.274 | 1.929 | -1.022 |
| BACS verbal memory | 0.727 | 0.763 | -0.523 | -0.578 | 0.178 | -1.016 | -0.022 |
| BACS verbal fluency | -0.062 | 0.785 | -0.149 | -1.482 | 0.391 | -0.228 | 0.363 |
| BACS digit sequencing | 0.602 | 0.56 | -1.008 | -0.604 | -0.291 | -0.111 | 1.008 |
| BACS token motor | 0.539 | 0.404 | -0.768 | -1.127 | -0.522 | -0.163 | -0.22 |
| BACS symbol coding | 0.133 | -0.55 | -0.659 | -1.163 | -0.116 | -0.989 | 0.567 |
| BACS tower of London | 0.468 | 0.18 | -0.997 | -0.953 | 0.802 | -0.096 | 0.009 |

**Table S16: Correlations between clinical measures and perfusion in the visual/fusiform cortex subregions in individuals with PSD (model A)**

|  | r-value | p-value | p_BH_-value |
| --- | --- | --- | --- |
| PANSS_postotal-hOc1 CBF | 0.098 | 0.026 | 0.146 |
| PANSS_postotal-hOc2 CBF | 0.103 | 0.019 | 0.143 |
| PANSS_postotal-hOc3 CBF | 0.003 | 0.946 | 0.952 |
| PANSS_postotal-MT CBF | 0.121 | 0.006 | 0.143 |
| PANSS_postotal-FG2 CBF | 0.087 | 0.048 | 0.193 |
| PANSS_postotal-FG3 CBF | 0.090 | 0.041 | 0.192 |
| PANSS_postotal-FG4 CBF | 0.102 | 0.020 | 0.143 |
| PANSS_negtotal-hOc1 CBF | 0.032 | 0.463 | 0.763 |
| PANSS_negtotal-hOc2 CBF | 0.023 | 0.609 | 0.878 |
| PANSS_negtotal-hOc3 CBF | -0.004 | 0.920 | 0.952 |
| PANSS_negtotal-MT CBF | 0.046 | 0.300 | 0.699 |
| PANSS_negtotal-FG2 CBF | 0.020 | 0.650 | 0.878 |
| PANSS_negtotal-FG3 CBF | 0.038 | 0.388 | 0.763 |
| PANSS_negtotal-FG4 CBF | 0.066 | 0.135 | 0.473 |
| MADRS_total-hOc1 CBF | 0.003 | 0.952 | 0.952 |
| MADRS_total-hOc2 CBF | -0.018 | 0.690 | 0.878 |
| MADRS_total-hOc3 CBF | -0.009 | 0.835 | 0.935 |
| MADRS_total-MT CBF | -0.023 | 0.604 | 0.878 |
| MADRS_total-FG2 CBF | 0.105 | 0.017 | 0.143 |
| MADRS_total-FG3 CBF | 0.033 | 0.453 | 0.763 |
| MADRS_total-FG4 CBF | 0.051 | 0.251 | 0.639 |
| YMRS_total-hOc1 CBF | 0.038 | 0.390 | 0.763 |
| YMRS_total-hOc2 CBF | 0.033 | 0.459 | 0.763 |
| YMRS_total-hOc3 CBF | -0.014 | 0.753 | 0.878 |
| YMRS_total-MT CBF | 0.063 | 0.157 | 0.488 |
| YMRS_total-FG2 CBF | 0.060 | 0.177 | 0.495 |
| YMRS_total-FG3 CBF | 0.019 | 0.665 | 0.878 |
| YMRS_total-FG4 CBF | 0.015 | 0.743 | 0.878 |

**Note:** CBF measures were harmonized for scanner differences. Combat-adjusted total gray matter CBF was included as covariate for the perfusion measures. p_BH_-value = Benjamini & Hochberg corrected p-value. FG1 and hOc4 had to be discarded because of minimum volume requirements for CBF extraction. Bold values indicate statistical significance at the p_BH_<0.05 level.

MADRS, Montgomery–Åsberg Depression Rating Scale; Negtotal, negative total; PANSS, Positive and Negative Syndrome Scale; Postotal, positive total; YMRS, Young Mania Rating Scale.

**Table S17: Correlations between clinical measures and perfusion in the visual/fusiform cortex subregions in individuals with PSD (model B)**

|  | r-value | p-value | p_BH_-value |
| --- | --- | --- | --- |
| PANSS_postotal-hOc1 CBF | 0.104 | 0.024 | 0.223 |
| PANSS_postotal-hOc2 CBF | 0.079 | 0.085 | 0.395 |
| PANSS_postotal-hOc3 CBF | -0.021 | 0.652 | 0.913 |
| PANSS_postotal-MT CBF | 0.13 | 0.005 | 0.132 |
| PANSS_postotal-FG2 CBF | 0.071 | 0.125 | 0.437 |
| PANSS_postotal-FG3 CBF | 0.043 | 0.349 | 0.665 |
| PANSS_postotal-FG4 CBF | 0.082 | 0.076 | 0.395 |
| PANSS_negtotal-hOc1 CBF | 0.049 | 0.293 | 0.665 |
| PANSS_negtotal-hOc2 CBF | 0.029 | 0.527 | 0.777 |
| PANSS_negtotal-hOc3 CBF | 0.007 | 0.875 | 0.922 |
| PANSS_negtotal-MT CBF | 0.054 | 0.239 | 0.657 |
| PANSS_negtotal-FG2 CBF | 0.032 | 0.494 | 0.768 |
| PANSS_negtotal-FG3 CBF | 0.041 | 0.372 | 0.665 |
| PANSS_negtotal-FG4 CBF | 0.085 | 0.065 | 0.395 |
| MADRS_total-hOc1 CBF | 0.041 | 0.38 | 0.665 |
| MADRS_total-hOc2 CBF | -0.003 | 0.946 | 0.946 |
| MADRS_total-hOc3 CBF | 0.008 | 0.871 | 0.922 |
| MADRS_total-MT CBF | 0.016 | 0.733 | 0.922 |
| MADRS_total-FG2 CBF | 0.111 | 0.016 | 0.223 |
| MADRS_total-FG3 CBF | 0.011 | 0.814 | 0.922 |
| MADRS_total-FG4 CBF | 0.041 | 0.371 | 0.665 |
| YMRS_total-hOc1 CBF | 0.052 | 0.258 | 0.657 |
| YMRS_total-hOc2 CBF | 0.036 | 0.436 | 0.718 |
| YMRS_total-hOc3 CBF | -0.019 | 0.685 | 0.914 |
| YMRS_total-MT CBF | 0.073 | 0.114 | 0.437 |
| YMRS_total-FG2 CBF | 0.057 | 0.218 | 0.657 |
| YMRS_total-FG3 CBF | -0.014 | 0.759 | 0.922 |
| YMRS_total-FG4 CBF | -0.006 | 0.889 | 0.922 |

**Note:** CBF measures were harmonized for scanner differences. Combat-adjusted total gray matter CBF, age and sex were included as covariates for the perfusion measures. p_BH_-value = Benjamini & Hochberg corrected p-value. FG1 and hOc4 had to be discarded because of minimum volume requirements for CBF extraction. Bold values indicate statistical significance at the p_BH_<0.05 level.

MADRS, Montgomery–Åsberg Depression Rating Scale; Negtotal, negative total; PANSS, Positive and Negative Syndrome Scale; Postotal, positive total; YMRS, Young Mania Rating Scale.

**Main results of rescaled ComBat-adjusted perfusion data**


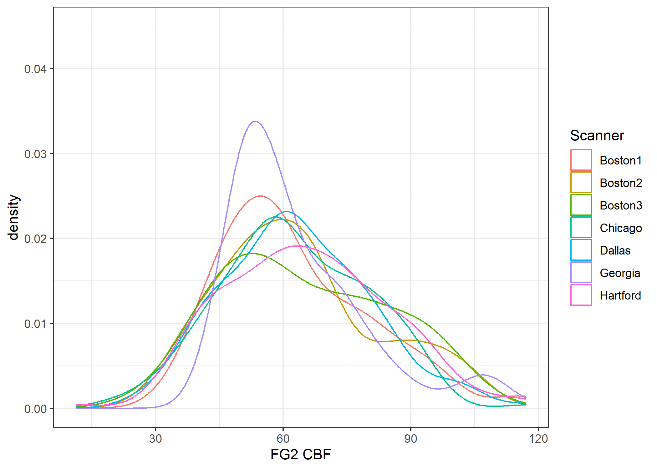

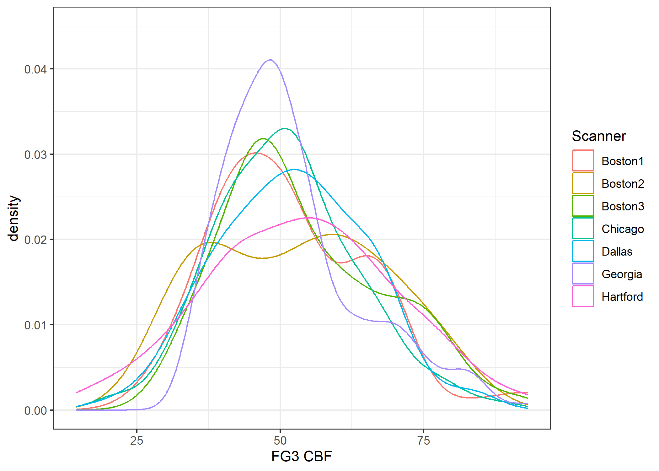

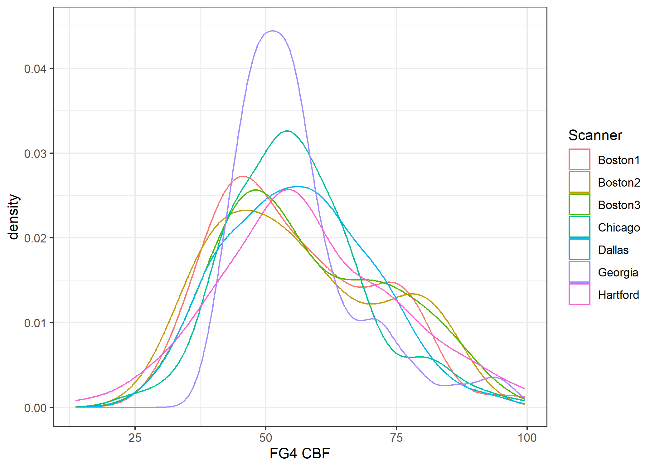

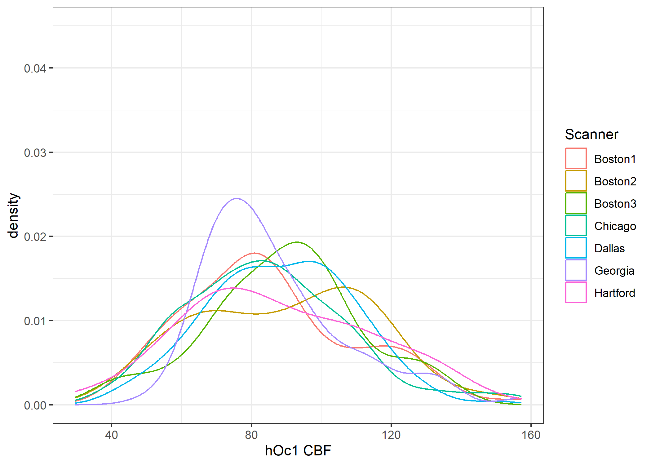

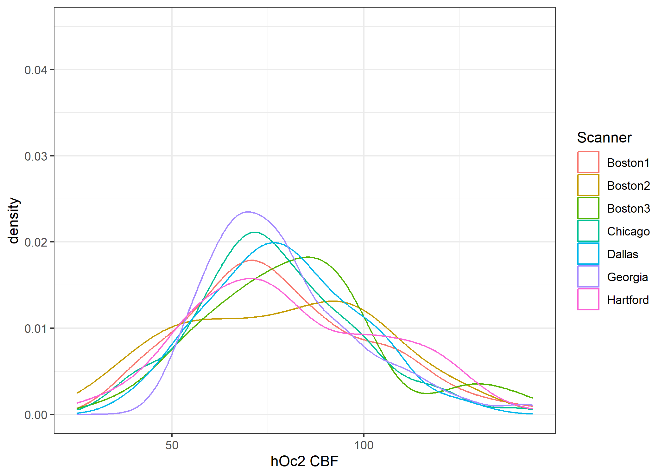

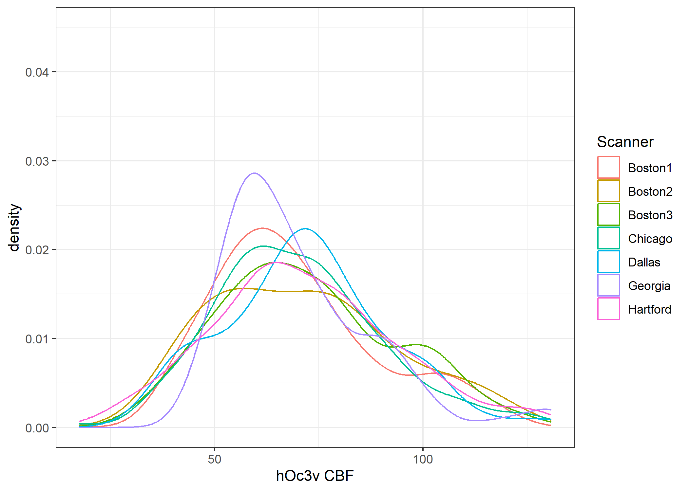

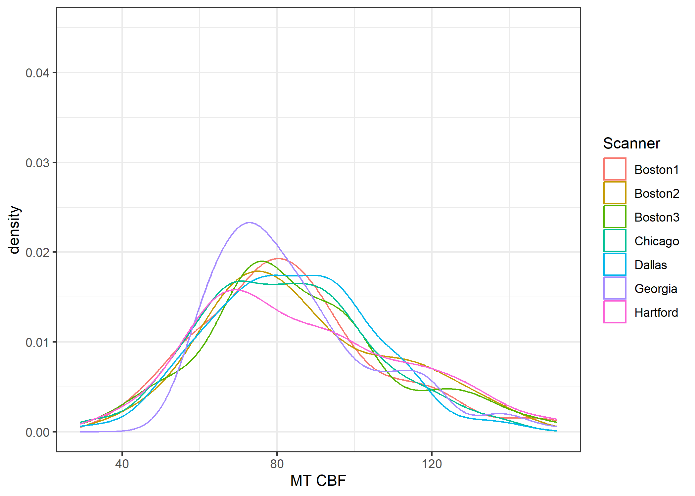


**Figure S5: Perfusion differences by site after outlier removal, winsorization, scanner harmonization and rescaling.** Density plots showing CBF density per region across scanners after outlier removal, winsorization, ComBat-adjustment and rescaling. Different colors represent different scanners. Significant scanner differences in the CBF data were eliminated in all regions.

**Figure S6.** **Cerebral blood flow (CBF) differences in MT and FG2 after rescaling perfusion values.** Boxplot with density plots demonstrating group differences between healthy controls (NC) and individuals with psychosis spectrum disorders (PSD) in MT **(A)** and FG2 **(B)**. MT and FG2 CBF were adapted for scanner-differences using ComBat and rescaled. CBF was covaried for ComBat-adjusted total gray matter (GM) CBF. Panel **(A)** shows significantly higher MT CBF in PSD compared with NC (p_BH_=0.014). Panel **(B)** demonstrates significantly greater FG2 CBF in PSD compared with NC (p_BH_=0.014).


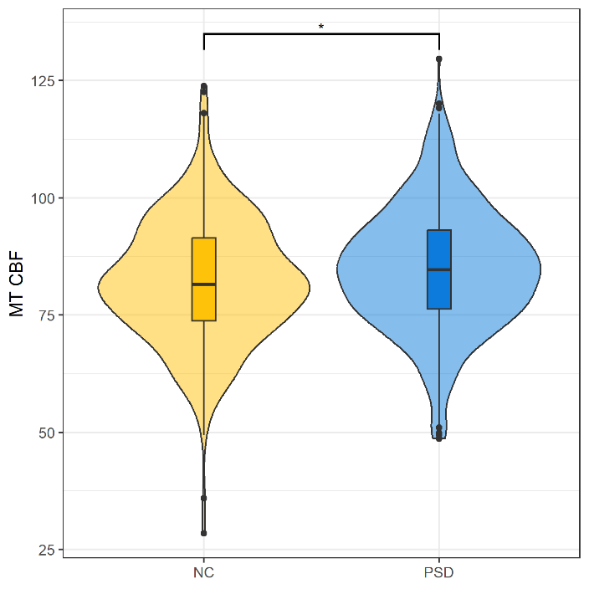

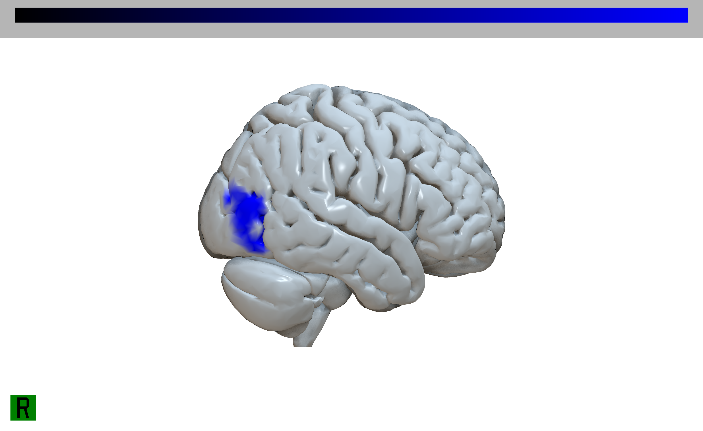


A


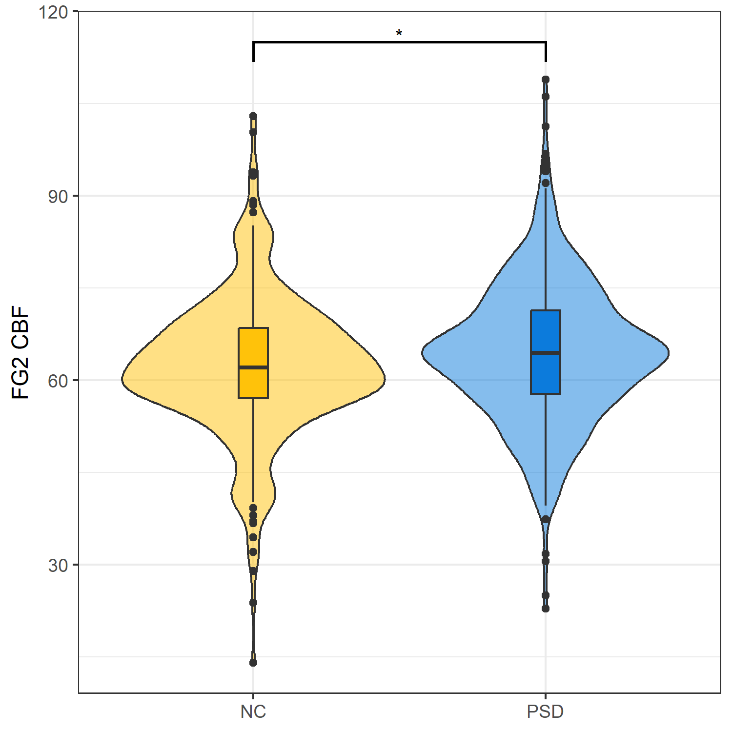

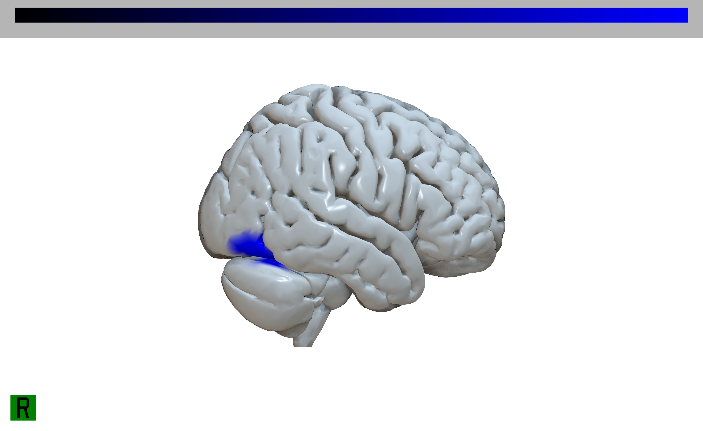


B


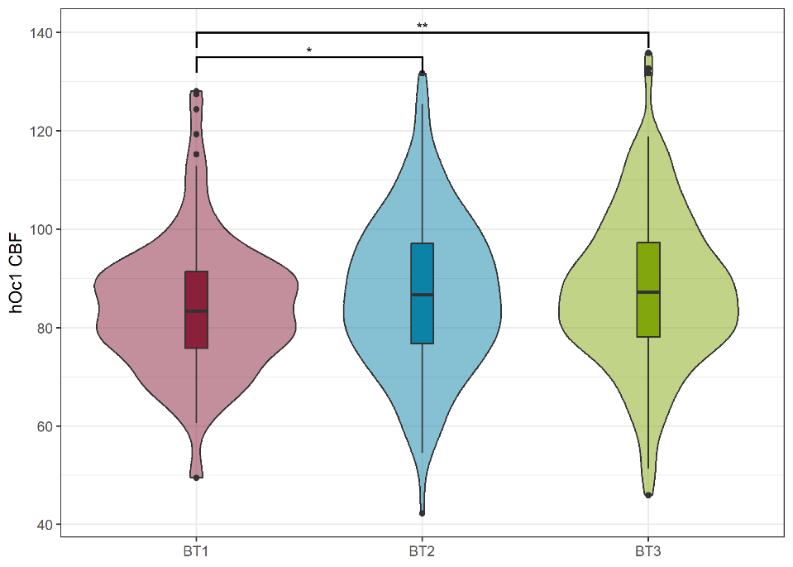


**Figure S7. Cerebral blood flow (CBF) differences across Biotypes in hOc1 after rescaling perfusion values.** CBF was adapted for scanner-differences via ComBat, rescaled and covaried for total gray matter (GM) CBF. There was significantly higher hOc1 CBF in BT2 (p_BH_=0.026) and BT3 compared to BT1 (p_BH_=0.001). Benjamini & Hochberg corrected p-values are denoted with “p_BH_”.

**Figure S8.** **Cerebral blood flow (CBF) correlation with cognitive measures in MT and FG4 after rescaling perfusion values.** Scatter plot in (A) showing partial Spearman correlations between scanner-adjusted and rescaled MT CBF and Brief Assessment of Cognition in Schizophrenia (BACS) composite score in individuals with psychosis spectrum disorders (PSD) and healthy controls (NC). There was a significant correlation between MT CBF and the BACS composite score in PSD (p_BH_=0.008). Scatter plot in (B) showing partial Spearman correlations between scanner-adjusted and rescaled FG4 CBF and BACS composite score in PSD and NC. There was a significant correlation between FG4 CBF and the BACS composite score in PSD (p_BH_=0.001). Total GM CBF was used as covariate for perfusion in all analyses.


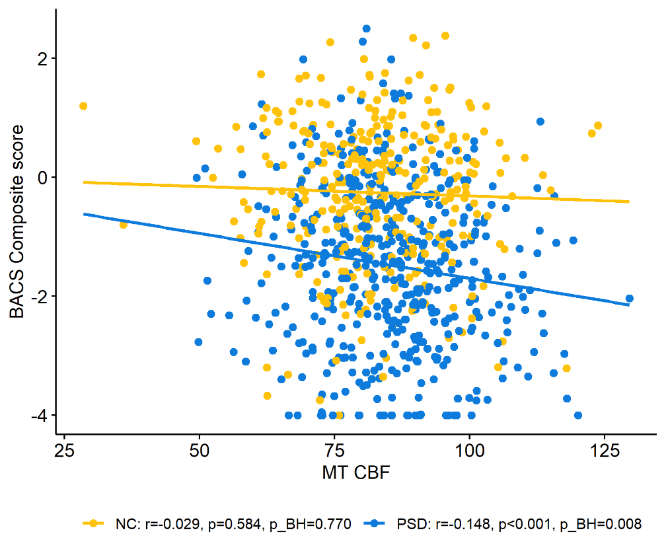

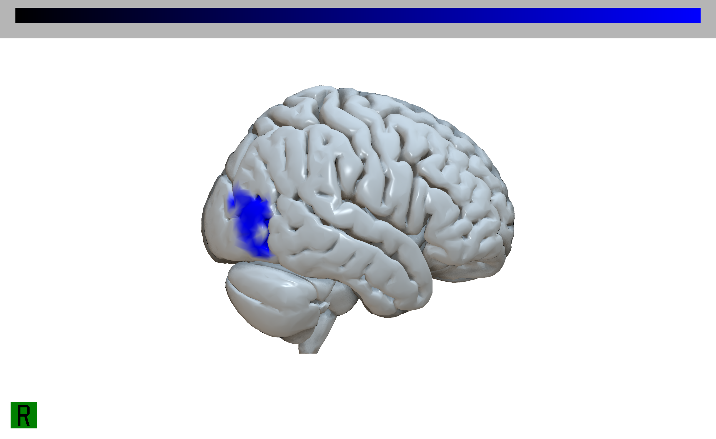


A


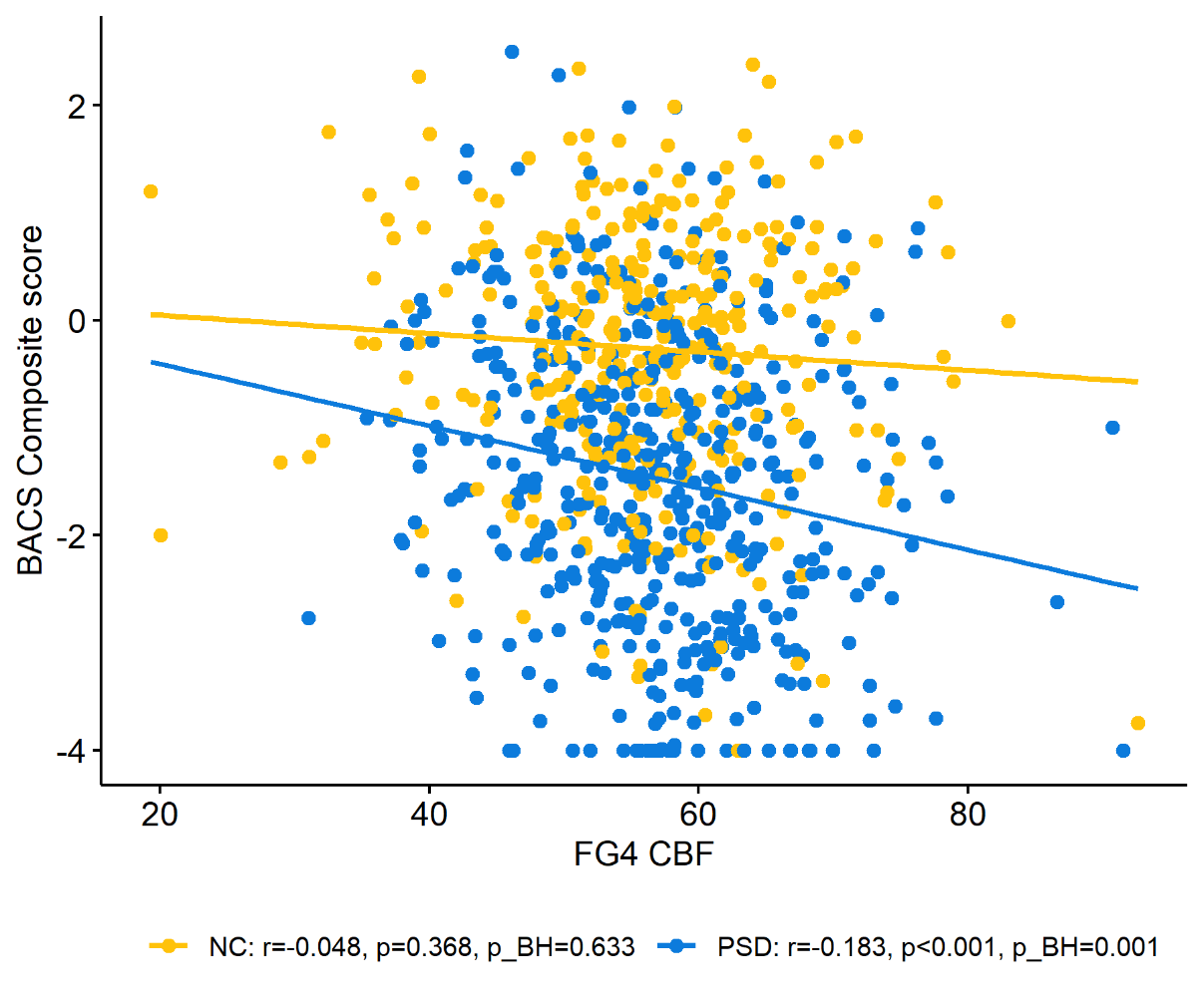

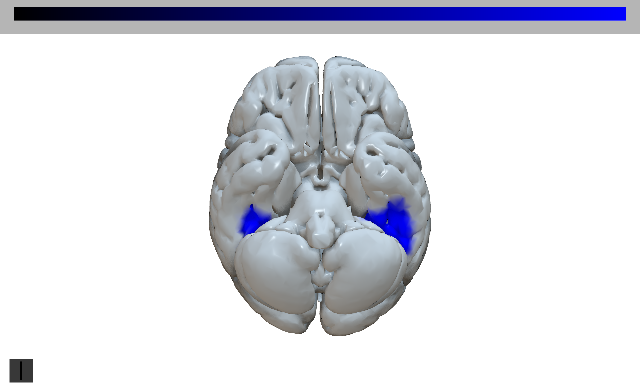


B

**References**

1. Tamminga CA, Pearlson G, Keshavan M, Sweeney J, Clementz B, Thaker G. Bipolar and Schizophrenia Network for Intermediate Phenotypes: Outcomes Across the Psychosis Continuum. *Schizophr Bull* (2014) 40:S131–S137. doi: 10.1093/schbul/sbt179

2. Clementz BA, Parker DA, Trotti RL, McDowell JE, Keedy SK, Keshavan MS, Pearlson GD, Gershon ES, Ivleva EI, Huang L-Y, et al. Psychosis Biotypes: Replication and Validation from the B-SNIP Consortium. *Schizophr Bull* (2022) 48:56–68. doi: 10.1093/schbul/sbab090

3. Tamminga CA, Clementz BA, Pearlson G, Keshavan M, Gershon ES, Ivleva EI, McDowell J, Meda SA, Keedy S, Calhoun VD, et al. Biotyping in psychosis: using multiple computational approaches with one data set. *Neuropsychopharmacology* (2021) 46:143–155. doi: 10.1038/s41386-020-00849-8

4. Clementz BA, Sweeney JA, Hamm JP, Ivleva EI, Ethridge LE, Pearlson GD, Keshavan MS, Tamminga CA. Identification of Distinct Psychosis Biotypes Using Brain-Based Biomarkers. *American Journal of Psychiatry* (2016) 173:373–384. doi: 10.1176/appi.ajp.2015.14091200

5. Desikan RS, Ségonne F, Fischl B, Quinn BT, Dickerson BC, Blacker D, Buckner RL, Dale AM, Maguire RP, Hyman BT, et al. An automated labeling system for subdividing the human cerebral cortex on MRI scans into gyral based regions of interest. *Neuroimage* (2006) 31:968–980. doi: 10.1016/j.neuroimage.2006.01.021

6. Türközer HB, Lizano P, Adhan I, Ivleva EI, Lutz O, Zeng V, Zeng A, Raymond N, Bannai D, Lee A, et al. Regional and Sex-Specific Alterations in the Visual Cortex of Individuals With Psychosis Spectrum Disorders. *Biol Psychiatry* (2022) 92:396–406. doi: 10.1016/j.biopsych.2022.03.023
